# Evolution and Stagnation of Image Guidance for Surgery in the Lateral Skull: A Systematic Review 1989–2020

**DOI:** 10.3389/fsurg.2020.604362

**Published:** 2021-01-11

**Authors:** Daniel Schneider, Jan Hermann, Fabian Mueller, Gabriela O'Toole Bom Braga, Lukas Anschuetz, Marco Caversaccio, Lutz Nolte, Stefan Weber, Thomas Klenzner

**Affiliations:** ^1^ARTORG Center for Biomedical Engineering, University of Bern, Bern, Switzerland; ^2^Department of Otorhinolaryngology, Head and Neck Surgery, Inselspital, University Hospital Bern, Bern, Switzerland; ^3^Department of Otorhinolaryngology, University Hospital Düsseldorf, Düsseldorf, Germany

**Keywords:** lateral skull, lateral skull base, neurotology, temporal bone, image-guidance, surgical navigation, accuracy, temporal evolution

## Abstract

**Objective:** Despite three decades of pre-clinical and clinical research into image guidance solutions as a more accurate and less invasive alternative for instrument and anatomy localization, translation into routine clinical practice for surgery in the lateral skull has not yet happened. The aim of this review is to identify challenges that need to be solved in order to provide image guidance solutions that are safe and beneficial for use during lateral skull surgery and to synthesize factors that facilitate the development of such solutions.

**Methods:** Literature search was conducted *via* PubMed using terms relating to image guidance and the lateral skull. Data extraction included the following variables: image guidance error, imaging resolution, image guidance system, tracking technology, registration method, study endpoints, clinical target application, and publication year. A subsequent search of FDA 510(k) database for identified image guidance systems and extraction of the year of approval, intended use, and indications for use was performed. The study objectives and endpoints were subdivided in three time phases and summarized. Furthermore, it was analyzed which factors correlated with the image guidance error. Factor values for which an error ≤0.5 mm (μ_error_ + 3σ_error_) was measured in more than one study were identified and inspected for time trends.

**Results:** A descriptive statistics-based summary of study objectives and findings separated in three time intervals is provided. The literature provides qualitative and quantitative evidence that image guidance systems must provide an accuracy ≤0.5 mm (μ_error_ + 3σ_error_) for their safe and beneficial application during surgery in the lateral skull. Spatial tracking accuracy and precision and medical image resolution both correlate with the image guidance accuracy, and all of them improved over the years. Tracking technology with accuracy ≤0.05 mm, computed tomography imaging with slice thickness ≤0.2 mm, and registration based on bone-anchored titanium fiducials are components that provide a sufficient setting for the development of sufficiently accurate image guidance.

**Conclusion:** Image guidance systems must reliably provide an accuracy ≤0.5 mm (μ_error_ + 3σ_error_) for their safe and beneficial use during surgery in the lateral skull. Advances in tracking and imaging technology contribute to the improvement of accuracy, eventually enabling the development and wide-scale adoption of image guidance solutions that can be used safely and beneficially during lateral skull surgery.

## Introduction

Microsurgical procedures in the lateral skull present a challenge for surgeons. The geometric scale of the anatomical structures in the lateral skull in the submillimeter range (1) requires surgeons to work at the limits of their visual and tactile capabilities. Instrument and anatomy localization are at the expense of invasiveness caused by exposing structures to be preserved and their use as orientation landmarks. In the absence of more precise and less invasive alternatives, surgery of lesions poses a considerable risk of iatrogenic morbidity (2, 3). Therefore, particularly in cases of benign tumors, the preservation of function and anatomical structures is often prioritized over surgical radicality. Consequentially and in combination with uncertainty in anatomy localization, pathological structures cannot be sufficiently exposed, contributing to high recurrence rates, for example, in cholesteatoma surgery (4).

Image guidance constitutes a technological solution for ensuring accurate anatomy and instrument localization. It drives the limits of surgery beyond what is possible by human visual and tactile perception alone, potentially improving the efficacy and the safety of procedures. Its value has pushed these systems into routine clinical practice in various medical fields such as orthopedics (5), neurosurgery (6), radio surgery (7), interventional oncology (8), and rhinology (9). Nonetheless, more than three decades after the introduction of frameless stereotaxy and numerous research applications, the technology is not routinely applied in lateral skull surgery.

The aim of this review is to identify challenges that need to be solved in order to provide image guidance solutions safe and beneficial for use during lateral skull surgery and to synthesize factors that facilitate the development of such solutions.

## Methods

### Search Strategy

Medical subject headings (MeSH) were used for electronic database searches from January 1, 1989 up to March 31, 2020. The search was conducted in English in MEDLINE and the COCHRANE Library with MeSH terms relating to image guidance (“Surgery, Computer-Assisted,” “Neuronavigation,” “Stereotaxic Techniques”) and the lateral skull (“Cranial Fossa, Middle,” “Cranial Fossa, Posterior,” “Temporal Bone,” “Otolaryngology”). The exact search term can be found in the attachment.

### Selection of Studies and Eligibility Criteria

**Figure 1:**
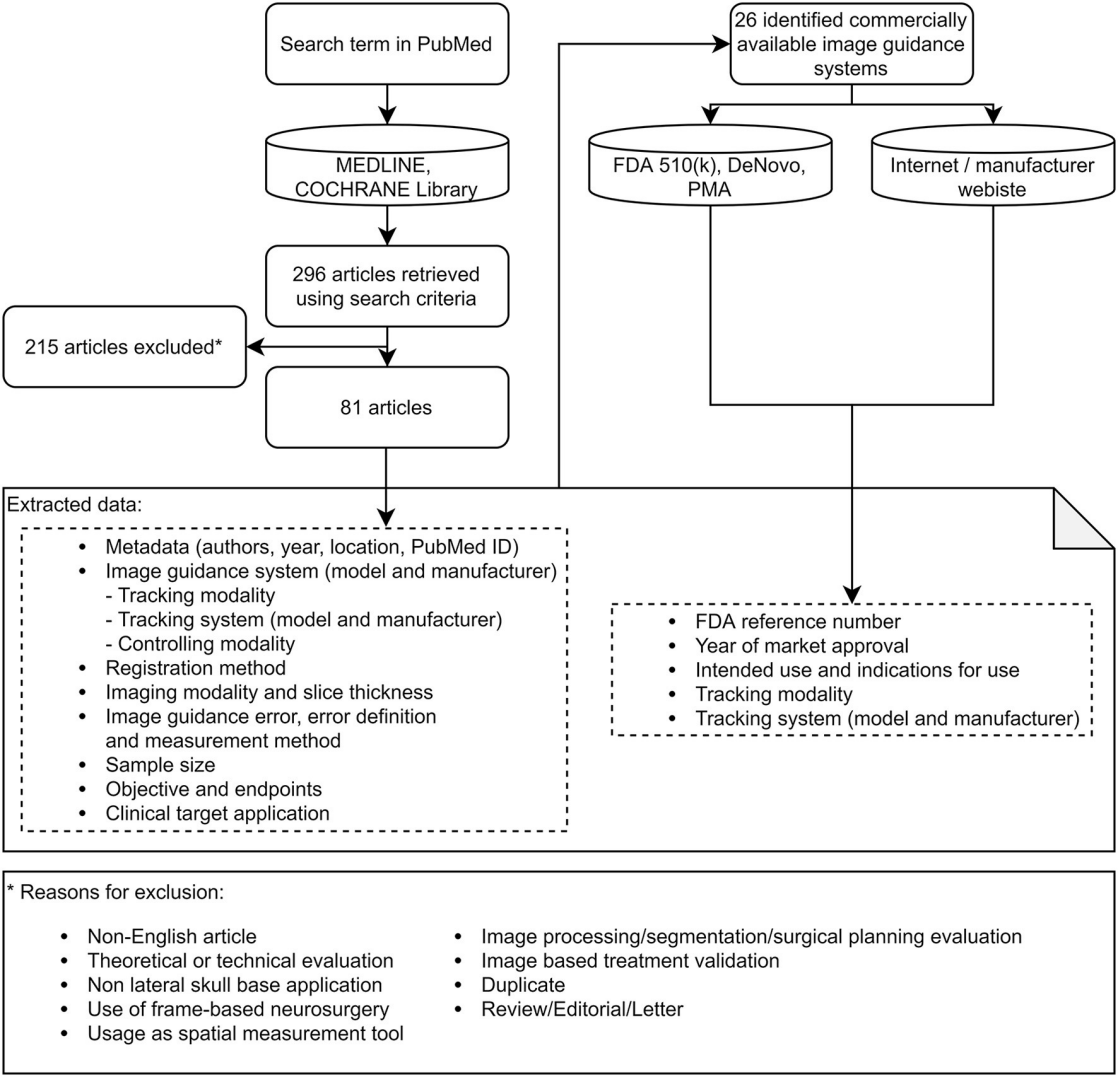
Search strategy and PRISMA data collection flow diagram.

A total of 296 articles were found. Subsequent exclusion of non-English-language articles to enable international verifiability, theoretical or technical evaluations, frame-based neurosurgery applications, non-lateral skull applications, applications where the system was purely used as distance measurement tool, image processing, segmentation, and surgical planning applications, articles about image-based treatment validation, duplicate articles, and review articles, letters, or editorials (Figure 1) resulted in 81 articles included in this review.

### Data Collection

For each article, the image guidance error (mean, standard deviation, error definition, and measurement method), imaging modality and slice thickness, tracking modality, tracking system manufacturer and model, instrument controller modality, image guidance system manufacturer and model, registration method, sample size, study objective, study endpoints, clinical target application and publication year, and other metadata were identified (Figure 1).

If the guidance error was reported using mean and maximum measured values, the reported maximum error was assumed to have happened with a probability of 0.5%; thus, the standard deviation was calculated as σ = (maximum value − μ)/2.5. For articles that did not report mean and standard deviation values but provided the measured errors, mean and standard deviation values were calculated. For articles that compared errors, the lowest error (μ_error_ + 3σ_error_) was considered. The error definitions were categorized into three- and two-dimensional position errors.

The tracking modalities were grouped as optical, electromagnetic, mechanical, acoustic, and no tracking. Instrument controlling modalities were grouped as freehand-controlled by the surgeon, robot-controlled, controlled through a stereotactic frame, controlled through a robotic stereotactic frame, and freehand-controlled drill by a surgeon with automatic on/off controller.

The clinical target applications were grouped into categories of cochlear implant surgery, internal auditory canal surgery, and general surgery in the lateral skull if otherwise.

The registration methods were grouped as anatomical landmark, skin-affixed fiducial, bone-anchored fiducial, skin surface-matching, bone surface-matching, dental bite block, template-assisted, and precalibrated imaging device registration.

For each identified image guidance system, the year of the first market approval (CE mark or FDA approval), the intended use, and the indications for use were identified by searching the FDA 510k, DeNovo, and PMA databases (10), which store information about cleared devices, on the official websites of the manufacturers or in press releases (Figure 1). For each system, the target market was identified by checking the marketing on the official webpage and grouped as “Lateral Skull” if the marketing targeted ear or lateral skull surgery and “Other” if otherwise.

### Data Analysis

To provide an overview of the research since 1989, market approvals and clinical studies were depicted in a timeline from 1989 to 2020. The study objectives and the endpoints were subdivided in three time phases and summarized.

The identified image guidance systems, tracking modalities and systems, controlling modalities, registration methods, clinical target applications, error measurement methods, and slice thickness values were visually assessed for differences in the image guidance error and values identified that were used in applications with an image guidance error ≤0.5 mm. The same factors and the image guidance error were assessed for correlation (Pearson) with the publication year, and any trends were identified. Additionally, use cases indicated in the indications for use and intended uses of the previously identified commercial image guidance systems were counted and consolidated into anatomical regions, and the respective proportions were calculated. Analysis was conducted in R (11), and the figures were produced using the package ggplot2 (12).

## Results

**Figure 2:**
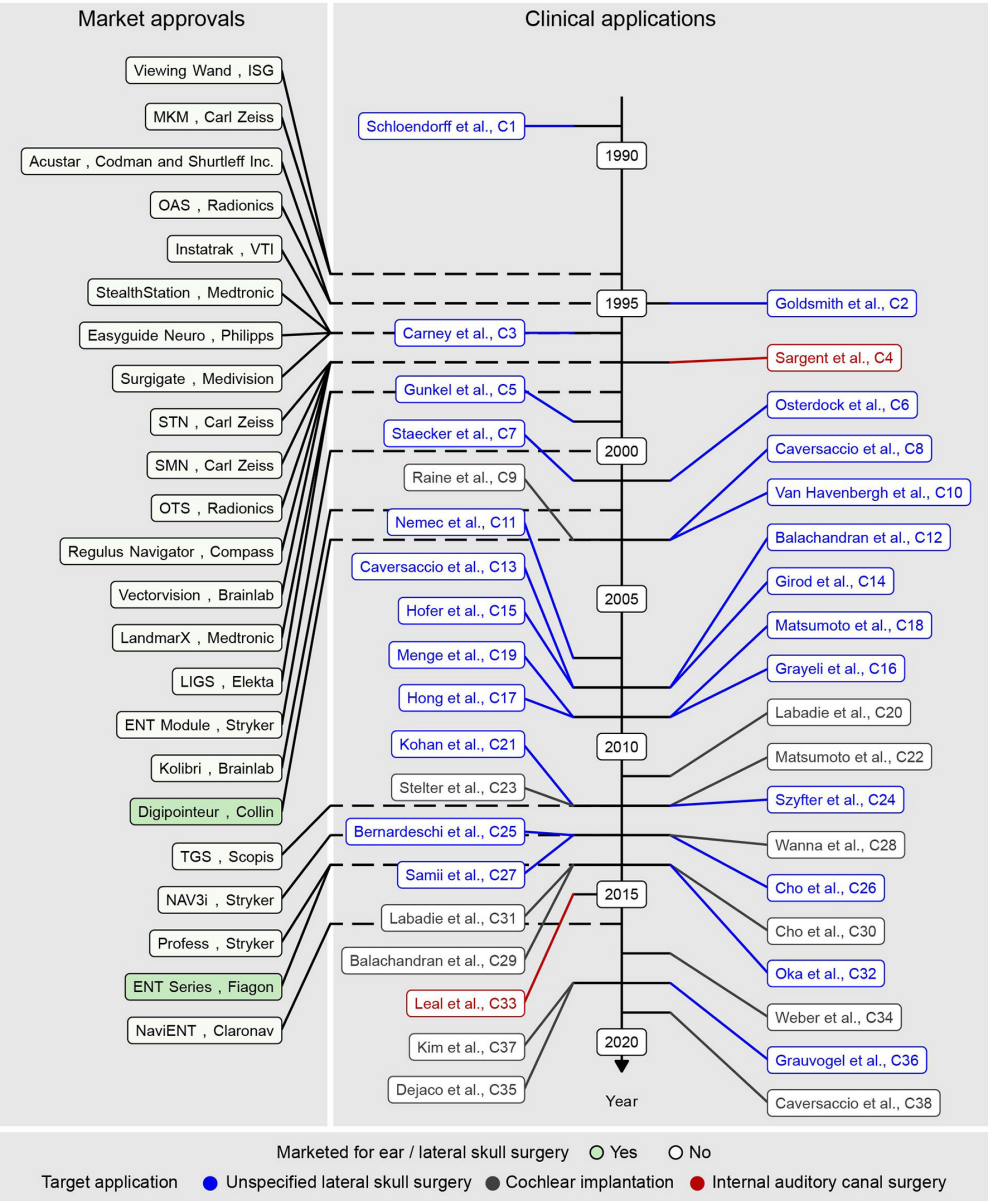
Market approval year of commercial image guidance systems (left) and clinical studies (middle, right) included in the present literature review in chronological order.

An overview of research conducted since 1989 highlighting the main findings is presented in the next three sections and in Figure 2. In the subsequent section, factors that allow the development of image guidance solutions that are safe and beneficial for use during surgery in the lateral skull are presented. In this context, image guidance components associated with low image guidance error and respective temporal evolution are presented. All included articles and extracted values can be found in the attachment in the form of a table and linked figures.

### 1989–2000: Identification of Clinical Use Cases and Envisionment of Positive Effects on Clinical Outcomes

The use of image guidance in lateral skull surgery was reported for the first time in 1989 by Schlöndorff et al. (13). A mechanical arm for spatial tracking, computed tomography (CT, slice thickness: 2 mm), and skin-affixed fiducial registration enabled the visualization of the arm-attached stylus location in the CT images on a computer (13). The system was applied in more than 100 ear, nose, and throat applications. Sinus, skull base, and tumor surgery were subjectively identified as applications that benefit from image guidance (14).

Early clinical image guidance applications in lateral skull surgery discussed the value of image guidance for different surgical applications. The technology was, albeit without significant evidence, reported as useful for the spatial identification of anatomy and pathology during surgical access creation to the petrous apex (15, 16) and the internal auditory canal (15–17), resection of (pseudo)neoplasms (15, 16, 18), and revision surgery (16). Reduced invasiveness (15–17) and iatrogenic morbidity (15–17) and increased efficacy (15, 16) of surgical treatments were envisioned. Advantages of non-over-mechanically linked systems (17), advantages of using a skull-attached patient tracker over patient fixation with a Mayfield clamp (16), advantages and disadvantages of different registration methods (19, 20), and adequately tracked instrumentation (15, 16) were also discussed. The routine applicability of available systems for microsurgery in the lateral skull was demonstrated (19).

By the year 2000, 16 image guidance systems from 11 companies were commercially available and cleared through the premarket notification [510(k)] process, stating their substantial equivalence to legally marketed predicate devices (Figure 2). Eleven, three, and two systems used infrared-based optical, mechanical, and electromagnetic instrument tracking, respectively. Among the optical tracking-based systems, nine systems used a passive Polaris camera (NDI, Canada), and the other two used an active Optotrak (NDI, Canada) and an active Stryker (Kalamazoo, USA) proprietary camera, respectively.

### 2000–2010: Technological Developments Without Clinical Progress

The clinical availability of image guidance systems resulted in multiple clinical studies and case reports investigating the clinical effects of image guidance. Despite the high expectations, no significant effect has yet been proven. Clinical applications reported few to no complications (21–25), complete tumor resection (23, 25), no tumor recurrence (25, 26), good audiological outcomes (21, 22), low number of required puncture attempts in otogenic brain abscess surgery (27), higher security and less stress for the surgeon (21, 22), and favorable reconstruction of the external ear canal (21), however without comparison to non-image-guided surgery. A decrease (16, 21) as well as an increase (26) in operating time was measured. Furthermore, image guidance was used to control the surgical burr motor and automatically stop the rotation in case of critical proximity to structures to be preserved; however, the anticipated decrease of complications during temporal bone drilling could not be proven (28). The status quo was similar to that of 10 years before: image guidance was reported as useful for identifying anatomy and pathology in pseudo(neoplasm) resection (22, 23, 25), cochlear implantation (24, 29), congenital aural atresia (21), and auricular implant (30) surgery, but no significant positive effects were demonstrated.

While sinus (9) and neurosurgery (6) benefited from the routine application of image guidance by 2010, the systems remained left out of routine clinical use in lateral skull surgery.

As between 2000 and 2010 the gap between promised effects and clinical reality became evident, an accuracy of commercially available systems of 1–3 mm (μ_error_ + 3σ_error_) was measured in independent studies (25, 31–34), and insufficient image guidance accuracy was identified as a reason (35–38). High costs in terms of additional radiation (29, 39), time consumption (26), and surgical invasiveness (40) contributing to the unfavorable cost–benefit ratio were also identified as decisive factors for driving the absence of these systems during lateral skull surgery. Even though the reduction of costs *via* non-invasive and quicker registration methods (40–42) was successful, the continued lack of accuracy limited operators from exploiting the benefits of image guidance in clinics.

### 2010–2020: Stagnation of Commercial Development, the Use Case of Cochlear Implantation, and the Fight for Microns

**Figure 3:**
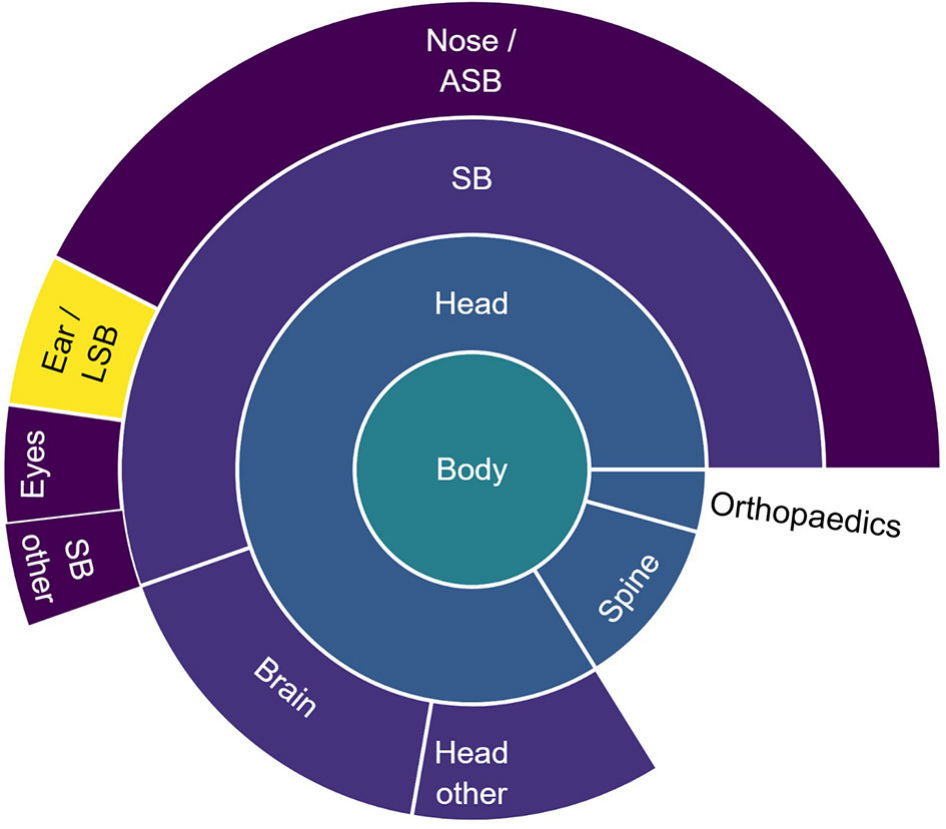
Proportion of the number of use cases for freehand image guidance per anatomical region. The use cases were extracted from the indications for use of commercial freehand image guidance systems used in the studies included in this literature review and were categorized into anatomical regions. SB, skull base; ASB, anterior skull base; LSB, latera skull base.

Between 2010 and 2020, the commercial development of image guidance for lateral skull surgery stagnated. During this period, five systems entered the market (Figure 2), including two systems from manufacturers who already invested in the market with other systems. All 23 systems identified in this study were cleared through the 510(k) route, indicating substantial equivalence to predicate devices. Overall, the available image guidance systems were and still are primarily developed and indicated for nose, anterior skull base, and neurosurgery (Figure 3).

**Figure 4:**
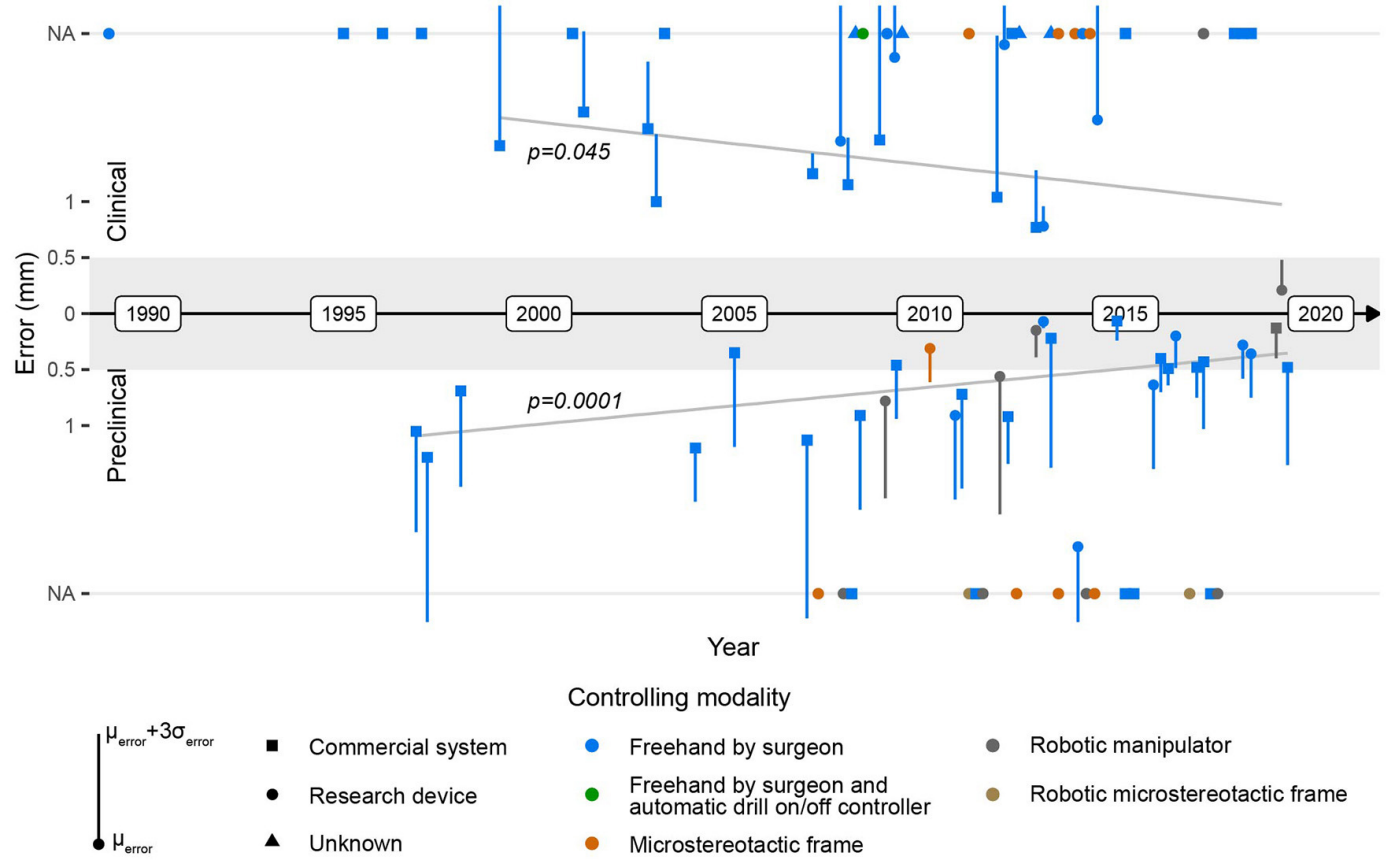
Evolution of the image guidance error in clinical (top) and preclinical cadaver (bottom) applications. The gray area depicts an error range ≤0.5 mm, and the gray lines depict the error trendlines. NA, value not available/reported.

Between 2010 and 2020, a large portion of research (25/50 included articles) focused on image-guided cochlear implantation. Image guidance technology conceptually enables the creation of a keyhole access tunnel from the mastoid surface to the middle and the inner ear which can be used for cochlear implantation, thus reducing the volume of bone removed. The practicability of creating a keyhole access route (diameter ~2 mm) to the middle ear with a freehand-controlled drill and relying on image guidance was investigated in preclinical experiments (43, 44) and found to be difficult and dangerous (45). Middle ear access creation for cochlear implantation requires drilling of bone between the facial nerve and chorda tympani [in-between distance: ~2.5 mm (46)]. To overcome human limitations in dexterity in creating keyhole access routes (45), the use of image-guided drill guides (45) (microstereotactic frames) and robotic manipulators (47, 48) was investigated. However, in preclinical studies, the lack of accuracy of both led to unacceptable rates of damage to the facial nerve, the chorda tympani, the ear canal wall, and the ossicles (47, 48). Empirical accuracy requirements of 0.5 mm (38) and 1 mm (24, 43) for safe application in cochlear implantation were discussed. However, it was only until recently and quantitative analysis (46) that the research community and medical device manufacturers started understanding the stringent maximum error requirements of ~≤0.5 mm (μ_error_ + 3σ_error_) needed for a safe, purely image-guided middle ear access creation in a large portion of the population with available and suitable instrumentation (drill diameter: 1–2 mm). Means to increase the accuracy of image guidance systems were investigated (49–54) with successful results (49, 50, 53) (Figure 4). Reliably accurate image guidance in combination with additional safety measures (55) has since enabled the successful clinical realization of the middle ear keyhole access (diameter: ~2 mm) using micro-stereotactic frames (56) and an image-guided robot (57) in seven of nine and six of nine patients, respectively. During the study with microstereotactic frames, one case was converted to conventional surgery and one case suffered from facial paresis (56). During the study with an image-guided robot, three cases were converted to conventional surgery (57). Safe operations and successful implantations in all patients but one demonstrated the safety and the feasibility of cochlear implantation through the associated keyhole approach. The clinical feasibility of this novel image-guided approach and the inherent benefits of reduced invasiveness, which cannot be exploited without image guidance, confirmed cochlear implantation as a use case for image guidance. However, the surgery time in these studies was substantially higher than in conventional surgery. Furthermore, the treatment caused additional radiation exposure to the patients and required additional personnel in the operating room (56, 57). Nevertheless, due to the promising clinical results and the potential benefits of the technology, a new market that has been targeted by multiple start-up companies (OtoJig, Germany; CAScination, Switzerland; Eindhoven Medical Robotics, Netherlands) has emerged.

Similar to those in previous decades, clinical applications of freehand image guidance in lateral skull procedures have suggested potential usefulness in otologic, petrous apex, and internal auditory canal surgery (58–61). Preclinical research has focused on finding accurate yet non-invasive (53, 54, 62) and automatic (42, 63) registration strategies, developing less-invasive access routes to the petrous apex and internal auditory canal (64–70) and assessing the accuracy of available technology (71, 72). The persistent lack of proven effects and absence in routine use indicate, however, that the clinically available systems still do not meet the needs for lateral skull surgery. State-of-the-art image guidance technology and research systems have reached an accuracy level (μ_error_ + 3σ_error_ ≤0.5 mm) that has been reported in the literature as a threshold for useful image guidance in the latera skull base. Commercialization of the technology is underway, paving the way for it to potentially become available to patients.

### Factors Facilitating the Development of Safe and Beneficial Image Guidance for Lateral Skull Surgery

The intended use of freehand image guidance systems is to precisely locate anatomical structures in surgery and, with this, potentially increase the safety and the efficacy of surgery. Insufficient accuracy of image guidance systems has been identified (35–38) as a decisive reason for their poor performance during lateral skull surgery and the consequential absence of positive effects on surgical safety and efficacy. The literature provides qualitative (38) and quantitative (46) evidence that an image guidance accuracy ≤0.5 mm (μ_error_ + 3σ_error_) is required for the safe and beneficial application of such technology in lateral skull surgery. Although the image guidance error continuously decreases over time (significant correlation, Pearson coefficients for clinical data: *p* = 0.045; preclinical data: *p* = 0.0001; combined: *p* = 4.1e-6), to date no commercial image guidance system that meets this requirement exists (Figure 4). However, during the last decade, a few research devices with an image guidance error ≤0.5 mm (μ_error_ + 3σ_error_) have been successfully developed, and their functionality and safety have been clinically validated, potentially suggesting their future beneficial application in lateral skull surgery (Figure 4).

To synthesize factors that allow a sufficiently accurate image-guidance, it was visually analyzed which factors allow low image-guidance error. Factor values for which an error ≤0.5 mm (μ_error_ + 3σ_error_) was measured in more than one study were inspected for time correlations.

**Figure 5:**
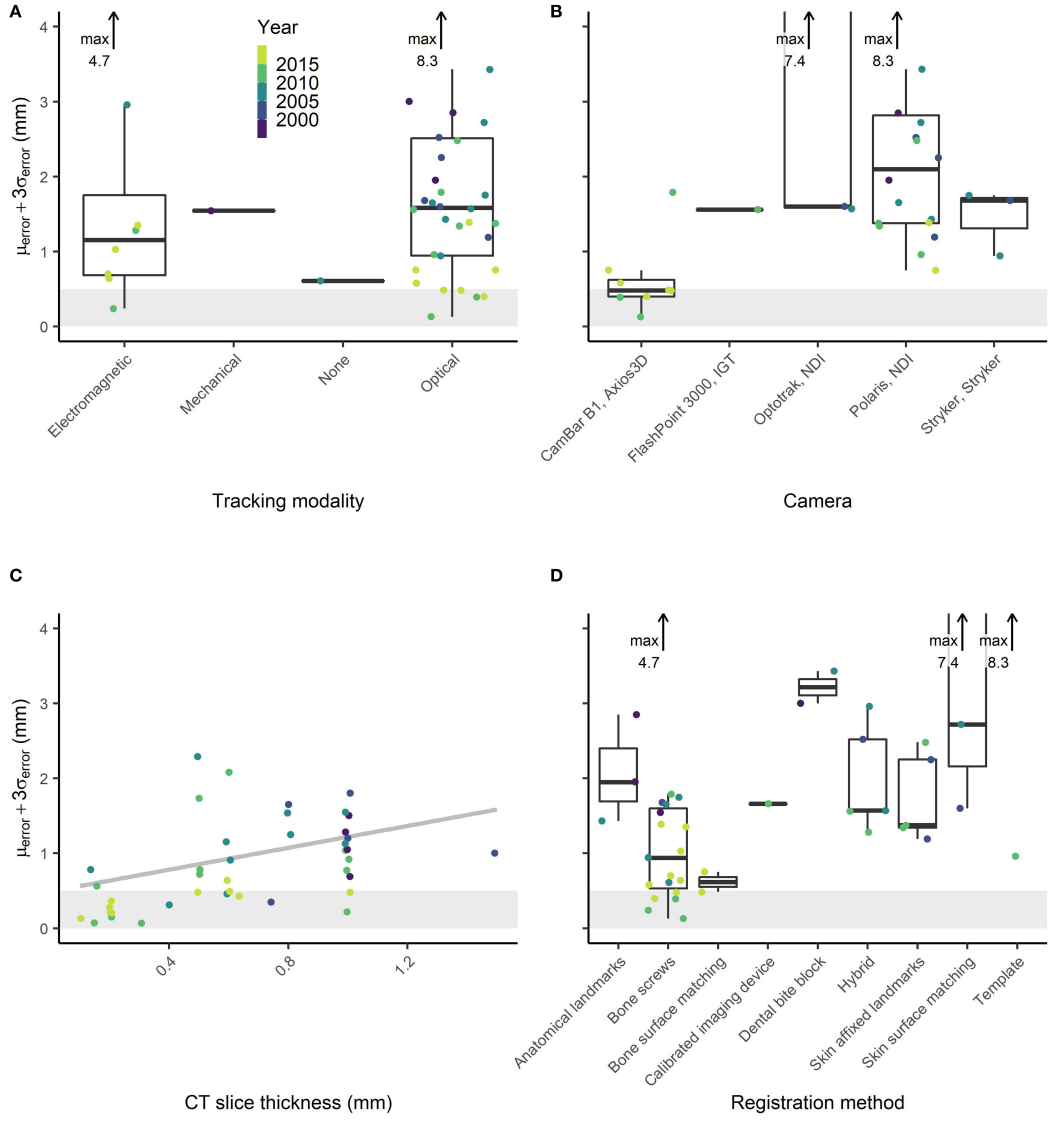
Reported image guidance errors for different **(A)** tracking modalities (none refers to stereotactic frames), **(B)** optical tracking cameras, **(C)** slice thickness values, and **(D)** registration methods.

**Figure 6:**
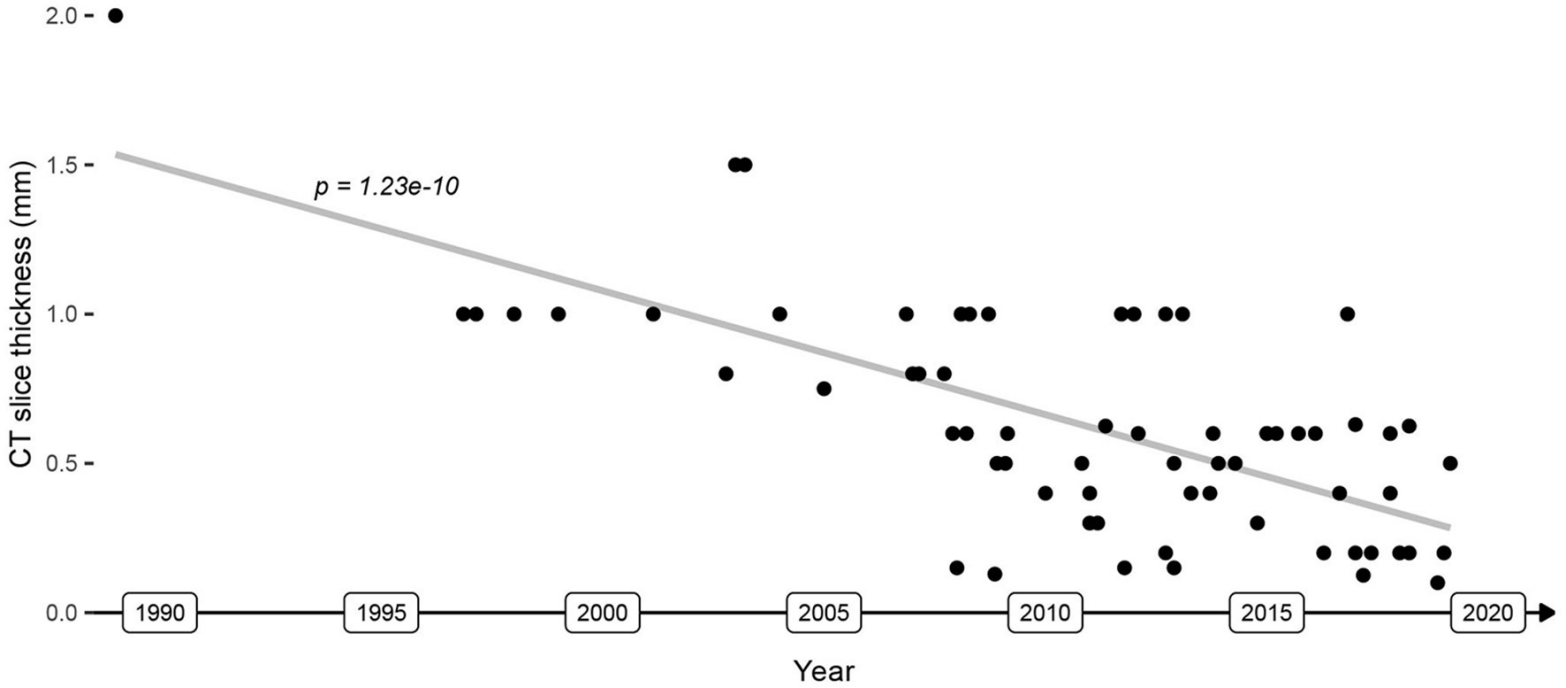
Evolution of CT image slice thickness used in image guidance applications. The gray line depicts the image resolution trendline.

Image guidance systems for which an error ≤0.5 mm (μ_error_ + 3σ_error_) was measured in more than one study conducted imaging with a slice thickness ≤0.2 mm, used optical tracking with a spatial localization accuracy and precision of ≤0.05 mm [P CamBar B1, Axios3d, Germany (73)], and used bone screw registration (Figure 5). The slice thickness of CT imaging decreased from 2 mm in 1989 to 0.1 mm in 2019 (Pearson coefficient *p* = 1.2e-10, Figure 6). A slice thickness ≤0.2 mm was uncommon before 2010 (2/25 studies which declared the used slice thickness). Also, until 2010, no study used optical tracking with spatial localization accuracy and precision ≤0.05 mm. The CamBar B1 optical tracking camera (Axios3D, Germany) has been marketed since 2010.

To answer the study objective, image guidance systems must reliably provide an accuracy ≤0.5 mm (μ_error_ + 3σ_error_) for their safe and beneficial use in lateral skull surgery. Instrument and patient tracking with an error ≤0.05 mm, CT imaging with a slice thickness ≤0.2 mm, and registration based on bone screws present components with which it is possible to develop clinically applicable and sufficiently accurate image guidance.

## Discussion

A clinical need for a non-invasive, anatomy-independent, and accurate means of anatomy localization and instrument navigation during lateral skull surgery has been reported from the early stages of standardized surgery in the lateral skull in the late nineteenth century and continuously since then (74). Methods have been sought in vain to reduce the risk of iatrogenic injury to anatomical structures, in particular, to the facial nerve (74). Over the last three decades, work on an image guidance-based solution has been carried out under continuous clinical evaluation. However, translation into routine clinical practice has not happened yet. Herein the major findings of three decades of research are summarized. Essential factors that will facilitate the development and wide-scale adoption for surgical use of image guidance solutions that are safe and beneficial for lateral skull surgery are synthesized and presented.

### Clinical Implications of the (Un)available Accuracy

The smallest and most delicate structures of the lateral skull are located in the temporal bone surrounding the labyrinth. Therefore, transmastoidal, transtympanic, and all transpetrosal access routes and surgery performed on the petrous bone embedding the internal auditory canal and labyrinth require high, yet clinically unavailable, levels of image guidance accuracy. As a consequence of the high recurrence rates after, the iatrogenic morbidity of conventional surgical treatments, and the technological progress in the domain of radiation therapy, temporal bone tumors are being irradiated with increasing frequency. Only recently the research community and medical device manufacturers have realized the stringent and technically challenging image guidance accuracy levels required to exploit the benefits of image guidance in large parts of the lateral skull. With state-of-the art imaging and tracking technology, the clinical feasibility of image guidance with suitable accuracy (μ_error_ + 3σ_error_ <0.5 mm) was demonstrated. Whether the ratio between the consequential benefits and the costs incurred favors, its application should be reassessed. Positive results would encourage translation of the technology into commercial systems and routine clinical practice and make it available to patients.

Transcranial access routes to the lateral skull base such as retrosigmoid or subtemporal access used to resect medium- to large-sized pathology, also extending into the brain from the internal auditory canal, clivus, or cerebellopontine angle, largely bypass the small temporal bone anatomy. As a consequence, a lower accuracy is needed to achieve the beneficial application of image guidance. Such procedures nowadays already benefit from commercially available systems, enabling the conduct of disease-tailored treatments through microscope injection of a surgical treatment plan that considers craniotomy size, access route, and target pathology. For this domain of neurosurgery, image guidance is advancing in terms of image processing algorithms, registration methods, visualization features, and usability and is therefore of increasing usefulness.

Furthermore, microsurgical procedures in the lateral skull are predominantly conducted using a microscope. Image-guided microscopic surgery requires focusing on either the microscope view or on the image guidance screen. Injection of the image guidance information into the microscope view potentially provides a solution but is subject to additional errors from microscope calibration.

To date no image guidance system can replace anatomical expertise, surgical training, or experience (16, 22, 25, 26, 31, 37).

### Accuracy Requirements

The accuracy requirements for image guidance in the lateral skull have been discussed various times, and maximum values of 0.5 mm (38) and 1 mm (16, 24, 35, 36) have been reported. The population size for which, depending on system accuracy and instrument diameter, keyhole access to the middle ear is safely realizable was quantitatively analyzed. With a system accuracy of 0.39 mm (μ_error_ + 3σ_error_) and a tool diameter of 1.8 mm, 47% of the adult population can be safely treated, indicating even stricter accuracy requirements (46). Similarly, the geometric size of the anatomical structures in the lateral skull (1), such as the chorda tympani diameter <0.5 mm, also indicates stricter accuracy requirements. The exact accuracy requirement values are, however, application and system specific. The accuracy requirement of 0.5 mm (μ_error_ + 3σ_error_) mentioned in this article is also referred to elsewhere in the literature (38) and is, in our opinion, an upper limit, below which the application of navigation becomes safe and its usefulness may exceed the “2nd-opinion” value.

### Risk of Bias in Error Definitions and Measurement Methodologies

The error of an image guidance system is defined as the spatial deviation of the instrument position reported by the system from its true position (75). The assessment of the quantitative error is susceptible to measurement bias where the measurement error corresponds to the degree of bias. To reduce the measurement bias during error assessments to a negligible level, the measurement error must be at least four times and preferably 10 times lower than the expected errors to be measured (76). For image guidance dedicated to lateral skull surgery, the measurement error that biases the measurement method must be ≲0.1 mm. To measure the image guidance error, the true spatial location of the instrument position at the time of measurement must be known. A common method (reported by 21/48 studies that declared their measurement methods) is to visually identify and manually select the position of an anatomical target landmark in the image data. Visual identification and manual selection of a landmark in the image data with a resolution of 0.1–1 mm are prone to errors, rendering this approach unsuitable for assessing the error of image guidance systems used for lateral skull surgery. As this is almost the only applicable assessment method in a clinical application [11/13 clinical applications, the other two used automatic detection of titanium target structures (34, 57)], effective error assessment is hardly possible in this case. The other 27 articles use, albeit unvalidated, more sophisticated error measurement methods. Validated means for preclinical and clinical assessments of the end-to-end error of freehand image guidance systems remain as unsolved challenges to date. Furthermore, the articles included in the review differ in terms of the definition and the measurement method of the image guidance error. The error measurement methods of the articles included in this review vary in target fiducials (24 × anatomical vs. 17 × artificial vs. 5 × unknown fiducials), identification of the true target position in image data (31 × manual vs. 10 × automatic vs. 5 × unknown identification), and spatial deviation measurement (8 × two-dimensional vs. 26 × three-dimensional vs. 12 × unknown error definition).

While the reported error may be sensible for the respective applications, due to different error definitions and measurement methods, the errors must be interpreted with caution. The risk of bias of each included study is indicated in the literature table in the attachment with low (artificial target, automatic ground truth target location identification), medium (artificial target, visual/manual ground truth target location identification), and high (anatomical target, visual/manual ground truth target location identification) measurement bias color-coded in green, orange, and red shades, respectively. A consequence of high measurement bias is that the error assessment of an image guidance system yields a quantitative estimate of the measurement bias rather than the image guidance error. The measured values overestimate the true image guidance error and are of limited value to draw conclusions about the systems error or determine potential improvements due to further development of a system. Since this study reports on trends and correlations and does not derive quantitative statistical error values from the collected errors, the measurement bias inherent in the source data is acceptable.

### Limitations

This literature review analyses correlations and time trends. Therefore, it is statistically impossible to imply causations. The use of adequate tracking technology, imaging protocols, and registration means does not necessarily lead to a system with sufficiently high accuracy as there might be other relevant factors that were not considered in this review, such as material and geometrical properties of the tracked instrumentation. Nevertheless, it was demonstrated (53, 55) that the use of these components allows the development of a clinically applicable system with sufficient image guidance accuracy for lateral skull surgery. The presented correlations and time trends provide evidence that the use of advanced tracking and imaging technology facilitates the development of a system with sufficient accuracy.

Surgery on the lateral skull includes a wide range of procedures. The submillimeter accuracy levels reported in the cited studies are only valid in bone and embedded structures. In soft tissue, the effective accuracy is much lower due to tissue shift. Therefore, the reported results are especially relevant for intra-temporal access routes and surgery, with access routes to and surgery in the lateral skull base being the most critical.

## Conclusion

After three decades of development and clinical application, no significant positive effects of image guidance on patient outcomes or other clinically relevant parameters in lateral skull surgery have been proven. Lack of available image guidance accuracy was identified by the research community as the predominant reason. Despite the prevailing need and long-known use cases, the existing commercially available systems are neither intended nor routinely used for lateral skull surgery.

Image guidance systems must reliably provide an accuracy ≤0.5 mm (μ_error_ + 3σ_error_) for their safe and beneficial use during surgery in the lateral skull. The CT slice thickness and the tracking accuracy improve continuously over time, which correlates positively with the temporal evolution of the image guidance error. The use of state-of-the-art spatial tracking with an error ≤0.05 mm, CT imaging with a slice thickness ≤0.2 mm, and bone-anchored titanium fiducials allows the development of image guidance systems with sufficient accuracy. While the translation into commercially available products and routine clinical practice has not happened yet, such technology has led to the development of image guidance systems with suitable accuracy for lateral skull surgery, and recent clinical evaluations have provided promising results, particularly in cochlear implantation.

## Data Availability Statement

The original contributions presented in the study are included in the article/supplementary materials, further inquiries can be directed to the corresponding author/s.

## Author Contributions

DS initiated the review, collected the data, reviewed data collected by JH and FM, conducted the data analysis, and wrote the manuscript. JH and FM assisted data collection, reviewed data collected by DS, reviewed the analysis, and reviewed the written manuscript. SW reviewed the data analysis and surveyed the literature review methodology. GB, LA, and MC reviewed the written manuscript. LN and TK contributed to the data collection, surveyed the literature review methodology, and reviewed the written manuscript. All authors contributed to the article and approved the submitted version.

## Conflict of Interest

SW is cofounder, shareholder, and chief executive officer of CAScination AG (Bern, Switzerland). The remaining authors declare that the research was conducted in the absence of any commercial or financial relationships that could be construed as a potential conflict of interest.
