# Supplementary material for: Evolution and Stagnation of Image Guidance for Surgery in the Lateral Skull: A Systematic Review 1989–2020
Source: Front Surg. 2021 Jan 11;7:604362. doi: 10.3389/fsurg.2020.604362 (PMC7831154; doi:10.3389/fsurg.2020.604362)
Supplement: Supplementary file 1 [file Data_Sheet_1.PDF]

ID	Year	First, last author	Objective	Endpoints	System type	Clinical target application	Error (mm) $\mu_{err}+3\sigma_{err}$ ($\mu_{err}\pm\sigma_{err}$)	Sample size	Error definition	Measurement method Risk of bias low: green, medium: orange, high: red, unknown: transparent	Imaging modality	Slice thickness (mm)	Navigation system model, company	Tracking system model, company	Tracking modality	Controlling modality	Registration method	Title	Place, Country	PMID
**Clinical application**																				
C1	1989	Schloendorff, Adams	To present a novel custom freehand image guidance system and discuss its value for ENT surgery	Use cases; Usefulness for positioning of radiation probes; Error	FIG	LSB	NA	100	NA	NA	CT	2	Custom, Custom	NA	Mechanical	Freehand by surgeon	Skin affixed landmarks	CAS - A new procedure in head and neck surgery	Aachen, Germany	2732101
C2	1995	Goldsmith, Nitsche	To discuss the value of commercial image guidance for providing minimally invasive treatment in neurotologic surgery	Use cases; Safety of surgery	FIG	LSB	NA	3	NA	NA	CT	NA	StealthStation, Medtronic	Polaris, NDI	Optical	Freehand by surgeon	Skin affixed landmarks	Clinical applications of frameless stereotactic devices in neurotology: preliminary report	Savannah, USA	8588648
C3	1996	Carney, Sandeman	To discuss the value of commercial image guidance for neurotologic surgery	Usefulness for identification of anatomy and pathology; Error; System associated time; System operability; Use cases	FIG	LSB	NA	14	NA	NA	CT	NA	Viewing Wand, ISG	NA	Mechanical	Freehand by surgeon	Skin surface matching	Intra-operative image guidance in otolaryngology - The use of the ISG viewing wand	Bristol, UK	8733452
C4	1997	Sargent, Bucholz	To discuss the value of commercial image guidance for tumor resection from the internal auditory canal	Error; System associated time; Usefulness for identification of anatomy; System operability	FIG	IAC	NA	NA	NA	NA	CT	NA	StealthStation, Medtronic	Polaris, NDI	Optical	Freehand by surgeon	Skin affixed landmarks	Middle cranial fossa surgery with image-guided instrumentation	St. Louis, USA	9230339
C5	1999	Gunkel, Freysinger	To assess the feasibility of routine application of commercial freehand image guidance in petrous bone surgery and to assess the systems accuracy	Error; Feasibility of dental bite block registration (VBH mouthpiece)	FIG	LSB	3 (1.5 ± 0.5)	11	Unknown	Anatomical target landmark, visual/manual identification of true position in image, manual digitization in patient with tracked instrument, manual/automatic deviation measurement between true and navigation-indicated position	CT	1	Viewing Wand, ISG	NA	Optical	Freehand by surgeon	Dental bite block	Computer-Aided Surgery in the Petrous Bone	Innsbruck, Austria	10569409
C6	2001	Osterdock, Crawford	To share experiences on the image guidance assisted (commercial freehand system) resection of a rare tumor	Description of symptoms, surgical resection treatment and outcomes; Usefulness for identification of anatomy and tumor extent; Usefulness for thorough tumor resection	FIG	LSB	NA	1	NA	NA	CT	NA	Cygnus PFS, Compass	3D Guidance trakSTAR, NDI	Optical	Freehand by surgeon	NA	Primary myxoma of the temporal bone in a 17-year-old boy: case report	New Orleans, USA	11322458
C7	2001	Staecker, Yoder	To assess the value of commercial freehand image guidance for lateral skull base surgery	Usefulness for identification of anatomy and pathology; Use cases; Usefulness for thorough tumor resection; Occurred complications; Error; System associated time	FIG	LSB	2.52 (1.8 ± 0.24)	11	Unknown	Anatomical target landmark, visual/manual identification of true position in image, manual digitization in patient with tracked instrument, manual/automatic deviation measurement between true and navigation-indicated position	CT, MRI	1 , 1	LandmarX, Medtronic	Polaris, NDI	Optical	Freehand by surgeon	Hybrid	Use of the LandmarX Surgical Navigation System in Lateral Skull Base and Temporal Bone Surgery	Baltimore, USA	17167627
C8	2003	Caversaccio, Haeusler	To share experiences on aural atresia surgery using image guidance and discuss the value of freehand image guidance for treatment	Surgery time (Image guided vs. not image guided); Usefulness for identification of anatomy; Usefulness for delineation of neo-canal direction; Audiological results; Error; Effects of system on surgeon (Stress, Confidence)	FIG	LSB	1.6 (1 ± 0.2)	18	3D positioning error	Anatomical target landmark, visual/manual identification of true position in image, manual digitization in patient with tracked instrument, manual/automatic deviation measurement between true and navigation-indicated position	CT	1.5	Surgigate, Medivision	Optotrak, NDI	Optical	Freehand by surgeon	Skin surface matching	Valuable use of computer-aided surgery in congenital bony aural atresia	Bern, Switzerland	12816210
C9	2003	Raine, Gopichandran	To present a freehand image guidance technique and discuss its value for cochlear implant surgery in the case of an ossified cochlea	Usefulness for identification of cochlear turns, facial nerve and internal carotid artery; System associated time; Radiation exposure	FIG	CI	NA	1	NA	NA	CT	1.5	LandmarX, Medtronic	Polaris, NDI	Optical	Freehand by surgeon	Anatomical landmarks	How We Do It: Using a surgical navigation system in the management of the ossified cochlea	Yorkshire, UK	18792141
C10	2003	Van Havenbergh, Verlooy	To assess the accuracy and value of commercial freehand image guidance in temporal bone surgery	System associated time; Error; Number of complete tumor resections; Number of tumor recurrence; Occurred complications	FIG	LSB	2.25 (1.65 ± 0.2)	2	3D positioning error	Anatomical target landmark, visual/manual identification of true position in image, manual digitization in patient with tracked instrument, manual/automatic deviation measurement between true and navigation-indicated position	CT	0.8	StealthStation, Medtronic	Polaris, NDI	Optical	Freehand by surgeon	Skin affixed landmarks	Image guided surgery for petrous apex lesions	Antwerp, Belgium	14505098
C11	2007	Nemec, Czerny	To assess the value of CT and MR image fusion for surgery planning and image guided (commercial freehand system) resection treatment of temporal bone tumors	Error; Efficacy of image fusion (Usefulness for delineation of tumor; Usefulness for differentiation between benign and malignant pathologies); Usefulness for identification of anatomy; Usefulness for avoiding iatrogenic injury; Occurred complications; Usefulness to achieve complete tumor resection	FIG	LSB	1.43 (1.25 ± 0.06)	15	Unknown	NA	CT, MRI	0.8 , 1	StealthStation, Medtronic	Polaris, NDI	Optical	Freehand by surgeon	Anatomical landmarks	CT-MR image data fusion for computer assisted navigated neurosurgery of temporal bone tumors	Vienna, Austria	17229539
C12	2008	Balachandran, Labadie	To present a method for clinical accuracy assessment of image guidance systems and thereby assess the accuracy of a commercial and a custom freehand image guidance systems for lateral skull base surgery	Error	FIG	LSB	3.43 (1.54 ± 0.63)	6	3D positioning error	Artificial target fiducial, automatic identification of true position in image, manual digitization in patient with tracked instrument, automatic deviation measurement between true and navigation-indicated position	CT	0.8	Custom, Custom	Polaris, NDI	Optical	Freehand by surgeon	Dental bite block	Accuracy of image-guided surgical systems at the lateral skull base as clinically assessed using bone-anchored hearing aid posts as surgical targets	Nashville, USA	18836389
C13	2008	Caversaccio, Haeusler	To assess the value of freehand image guidance for cholesterol granuloma drainage	System associated time; Usefulness for identification of anatomy; Occurred complications; Error; System operability; Effects of system on surgeon (Stress, Confidence)	FIG	LSB	1.57 (1.15 ± 0.14)	6	Unknown	Anatomical target landmark, visual/manual identification of true position in image, manual digitization in patient with tracked instrument, manual/automatic deviation measurement between true and navigation-indicated position	CT	0.6	Surgigate, Medivision	Optotrak, NDI	Optical	Freehand by surgeon	Hybrid	Cholesterol granuloma of the petrous apex: benefit of computer-aided surgery	Bern, Switzerland	18542981
C14	2008	Girod, Maurer	To discuss the value of image guidance for auricular reconstruction surgery	Usefulness for positioning of implant; Usefulness to avoid skull penetration; Usefulness to avoid iatrogenic injury	FIG	LSB	NA	1	NA	NA	CT	1	NA	NA	Optical	Freehand by surgeon	Skin affixed landmarks	Image-guided surgical navigation in implant-based auricular reconstruction	Stanford, USA	18486803
C15	2008	Hofer, Strauss	To assess the value of a custom image guidance controlled drill (drill on/off controller) for lateral skull base surgery	System associated time; Number of complications with image guidance vs. without image guidance; Effects of system on surgeon (Cognitive load)	NCD	LSB	NA	4	NA	NA	CT	1	Custom, Custom	Polaris, NDI	Optical	Freehand by surgeon and automatic drill on/off controller	Bone screws	First clinical evaluation of the navigated controlled drill at the lateral skull base	Leipzig, Germany	18391280
C16	2009	Grayeli, Sterkers	To assess the accuracy of commercial freehand image guidance and hybrid skin surface matching and bone screw registration for temporal bone surgery	Error	FIG	LSB	2.96 (1.55 ± 0.47)	33	3D positioning error	Anatomical target landmark, visual/manual identification of true position in image, manual digitization in patient with tracked instrument, manual/automatic deviation measurement between true and navigation-indicated position	CT	1	Digipointeur, Collin	Collin, Collin	Electromagnetic	Freehand by surgeon	Hybrid	Use of anatomic or invasive markers in association with skin surface registration in image-guided surgery of the temporal bone	Paris, France	19153848
C17	2009	Hong, Hashizume	To assess the accuracy and feasibility of a custom freehand image guidance system that supports patient motion compensation, template assisted anatomical landmark registration and real-time intraoperative CT image modification for image guided otologic surgery	Error; System operability; Occurred complications; Number of successful insertions	FIG	LSB	NA	5	NA	NA	CT	0.5	Custom, Custom	Polaris, NDI	Optical	Freehand by surgeon	Skin surface matching	Medical Navigation System for Otologic Surgery Based on Hybrid Registration and Virtual Intraoperative Computed Tomography	Fukuoka, Japan	19272886
C18	2009	Matsumoto, Komune	To assess the accuracy and feasibility of a template assisted anatomical landmark registration for image guided otologic surgery	Error; Feasibility of template assisted registration	FIG	LSB	6.94 (2.29 ± 1.55)	6	3D positioning error	Anatomical target landmark, visual/manual identification of true position in image, manual digitization in patient with tracked instrument, manual/automatic deviation measurement between true and navigation-indicated position	CT	0.5	Custom, Custom	Polaris, NDI	Optical	Freehand by surgeon	Template	A minimally invasive registration method using Surface Template-Assisted Marker Positioning (STAMP) for image-guided otologic surgery	Fukuoka, Japan	19130970
C19	2009	Menge, Pfeiffer	To share experiences on the image guidance assisted (commercial freehand system) resection of a rare tumor	Tumor recurrence; Occurred complications	FIG	LSB	NA	1	NA	NA	CT	NA	NA	NA	NA	Freehand by surgeon	NA	Giant cell reparative granuloma of the temporal bone	Freiburg, Germany	19224123
C20	2011	Labadie, Fitzpatrick	To assess the feasibility of cochlear keyhole access creation with custom microstereotactic frames	Clinical feasibility of microstereotactic frame manufacturing, processing and sterilization; Distances of drill hole axis to structures to be preserved; Potential successrate of safe facial recess drilling with different drill diameters	SF	CI	NA	18	NA	NA	CT	0.3	Custom, Custom	NA	None	Microstereotactic frame	Bone screws	Clinical Validation Study of Percutaneous Cochlear Access Using Patient Customized Micro-Stereotactic Frames	Nashville, USA	20019561
C21	2012	Kohan, Jethanamest	To assess the value of commercial freehand image guidance in otologic surgery	System associated time; Number of occurred complications; Error; System operability	FIG	LSB	2.48 (1.04 ± 0.48)	15	Unknown	Anatomical target landmark, visual/manual identification of true position in image, manual digitization in patient with tracked instrument, manual/automatic deviation measurement between true and navigation-indicated position	CT	1	LandmarX, Medtronic	Polaris, NDI	Optical	Freehand by surgeon	Skin affixed landmarks	Image-Guided Surgical Navigation in Otology	New York, USA	22961537
C22	2012	Matsumoto, Komune	To assess the feasibility and value of template assisted anatomical landmark registration for routine use in cochlear implant surgery	Error; System associated time; Number and description of occurred complications	FIG	CI	7.82 (2.4 ± 1.81)	25	3D positioning error	Anatomical target landmark, visual/manual identification of true position in image, manual digitization in patient with tracked instrument, manual/automatic deviation measurement between true and navigation-indicated position	CT	NA	Custom, Custom	Polaris, NDI	Optical	Freehand by surgeon	Template	Cochlear Implantation Assisted by Noninvasive Image Guidance	Fukuoka, Japan	22975902
C23	2012	Stelter, Mueller	To assess the feasibility and value of commercial freehand image guidance and automatic calibrated intraoperative CT based registration for cochlear implant surgery	Error; Usefulness for identification of cochleostomy location; Number of occurred complications; Audiological results; System associated time	FIG	CI	NA	1	NA	NA	CT	0.6	Vectorvision, Brainlab	Polaris, NDI	Optical	Freehand by surgeon	Calibrated imaging device	Image guided navigation by intraoperative CT scan for cochlear implantation	Munich, Germany	22452534
C24	2012	Szyfter, Bartochowska	To assess therapeutic outcomes of patients with otogenic brain abscess, one part of which was treated with image guidance assistance (commercial freehand system)	Mortality rates; Abscess locations; Occurred complications; Usefulness of image guidance for identification of puncture location of abscess	FIG	LSB	NA	5	NA	NA	NA	NA	NA	NA	NA	Freehand by surgeon	NA	Evolution in Management of Otogenic Brain Abscess	Poznan, Poland	22334160
C25	2013	Bernardeschi, Sterkers	To assess the accuracy of commercial freehand image guidance and a custom patient tracker for lateral skull base surgery	System associated time; Error; System operability (Stability of patient tracker)	FIG	LSB	1.28 (0.77 ± 0.17)	9	Unknown	Anatomical target landmark, visual/manual identification of true position in image, manual digitization in patient with tracked instrument, manual/automatic deviation measurement between true and navigation-indicated position	CT	1	Digipointeur, Collin	Collin, Collin	Electromagnetic	Freehand by surgeon	Hybrid	Use of bone anchoring device in electromagnetic computer-assisted navigation in lateral skull base surgery	Paris, France	23941593
C26	2013	Cho, Hashizume	To assess the value of custom freehand image guidance and auditory feedback on unsafe zones for otologic surgery	Error; Operability of system (Operability of auditory feedback); Usefulness for receiving image guidance feedback without removing visual focus from surgical site	FIG	LSB	0.96 (0.78 ± 0.06)	3	3D positioning error	Anatomical target landmark, visual/manual identification of true position in image, manual digitization in patient with tracked instrument, manual/automatic deviation measurement between true and navigation-indicated position	CT	0.5	Custom, Custom	Polaris, NDI	Optical	Freehand by surgeon	Template	Warning navigation system using real-time safe region monitoring for otologic surgery	Fukuoka, Japan	23161169
C27	2013	Samii, Gergonov	To share experiences on image guidance assisted (commercial freehand system) tumor resection through a hearing-preserving retrosigmoid approach	Tumor resection extent; Usefulness for identification of anatomy	FIG	LSB	NA	1	NA	NA	CT	NA	NA	NA	NA	Freehand by surgeon	NA	Retrosigmoid intradural inframeatal approach: indications and technique	Hannover, Germany	23361321
C28	2013	Wanna, Balachandran	To share experiences on the use of microstereotactic frames for cochlear implant surgery in the case of an ossified cochlea	Occurred complications; Surgery time; Feasibility of implant insertion; Insertion depth (full/partial)	SF	CI	NA	1	NA	NA	CT	NA	Custom, Custom	NA	None	Microstereotactic frame	Bone screws	Implantation of the completely ossified cochlea: An image-guided approach	Nashville, USA	23370556
C29	2014	Balachandran, Labadie	To assess the feasibility of cochlear keyhole access creation with custom microstereotactic frames in pediatric patients	Feasibility of system for pediatric patients (Number of collision-free plannable linear paths to cochlea through facial recess; Number of cases in which the 1mm diameter drill passes through the facial recess without contact to the facial or chorda tympani nerve); Distances of drill hole to structures to be preserved; Usefulness for identifying the cochleostomy location in cases with inner ear malformations	SF	CI	NA	13	NA	NA	CT	0.4	Custom, Custom	NA	None	Microstereotactic frame	Bone screws	Minimally-Invasive, Image-Guided Cochlear Implantation for Pediatric Patients â€“ Clinical Feasibility Study	Nashville, USA	18836389
C30	2014	Cho, Hashizume	To assess the feasibility of creating a cochleostomy with a custom freehand image guidance system with auditory feedback	Operability of system (Operability of auditory feedback); Usefulness of receiving image guidance feedback without removing visual focus from surgical site	FIG	CI	NA	2	NA	NA	CT	0.5	Custom, Custom	Polaris, NDI	Optical	Freehand by surgeon	Template	A Surgical Navigation System for Guiding Exact Cochleostomy Using Auditory Feedback: A Clinical Feasibility Study	Fukuoka, Japan	25093182
C31	2014	Labadie, Fitzpatrick	To assess the feasibility of cochlear keyhole access creation with custom microstereotactic frames and the associated keyhole approach for cochlear implantation	Number of completed access tunnels to the middle ear; Number of scala tympani insertions; Occurred extra- and intracochlear complications; Surgery time	SF	CI	NA	9	NA	NA	CT	NA	Custom, Custom	NA	None	Microstereotactic frame	Bone screws	Minimally-Invasive, Image-Guided Cochlear Implantation Surgery: First report of clinical implementation	Nashville, USA	24272427
C32	2014	Oka, Hashizume	To assess the accuracy, registration time and usability of precalibrated template assisted anatomical landmark registration for temporal bone surgery and compare it to its predecessor	System associated time; Error	FIG	LSB	8.3 (1.73 ± 2.19)	4	3D positioning error	Anatomical target landmark, visual/manual identification of true position in image, manual digitization in patient with tracked instrument, manual/automatic deviation measurement between true and navigation-indicated position	CT	0.5	Custom, Custom	Polaris, NDI	Optical	Freehand by surgeon	Template	A preregistered STAMP method for image-guided temporal bone surgery	Fukuoka, Japan	23801450
C33	2015	Leal, Ramina	To assess the value of commercial freehand image guidance for, and the feasibility of achieving adequate exposure for tumor resection from the internal auditory canal through an image guided labyrinth-preserving retrosigmoid approach	Feasibility of image guided surgical approach; Occurred complications	FIG	IAC	NA	30	NA	NA	CT	0.6	NA	Polaris, NDI	Optical	Freehand by surgeon	NA	Surgical exposure of the internal auditory canal through the retrosigmoid approach with semicircular canals anatomical preservation	Curitiba, Brazil	26017209
C34	2017	Weber, Caversaccio	To present an image guided robotic treatment model for cochlear implant surgery, its application to custom image guidance technology, and its application in a first patient	Description of robotic treatment model for cochlear implantation and its implementation with custom image guidance technology; Feasibility in a first patient (Feasibility of middle ear access creation and implant insertion; Insertion depth (degrees, number of intracochlear electrode contacts); Audiological outcomes; Occurred complications)	R	CI	NA	1	NA	NA	CT	0.2	Custom, Custom	CamBar B1, Axios3D	Optical	Robotic manipulator	Bone screws	Instrument flight to the inner ear	Bern, Switzerland	30246168
C35	2018	Dejaco, Schmutzhard	To share experiences on the use of commercial freehand image guidance for cochlear implant surgery in the case of an ossified cochlea	Insertion depth (full/partial; Audiological results); Number of occurred complications	FIG	CI	NA	1	NA	NA	CT	0.4	StealthStation, Medtronic	NA	Electromagnetic	Freehand by surgeon	Bone screws	Successful Cochlear Implantation of a Split Electrode Array in a Patient With Far-advanced Otosclerosis Assisted by Electromagnetic Navigation: A Case Report	Innsbruck, Austria	29995006
C36	2018	Grauvogel, Vasilikos	To share experiences on the use of commercial freehand image guidance for recurrent petrous apex cholesteatoma	Usefulness for identification of anatomy and pathology; Tumor resection extent	FIG	LSB	NA	1	NA	NA	MRI, CT	NA	iNtellect, Stryker	Stryker, Stryker	Optical	Freehand by surgeon	Skin affixed landmarks	Piezosurgery-, neuroendoscopy-, and neuronavigation-assisted intracranial approach for removal of a recurrent petrous apex cholesteatoma: technical note	Freiburg, Germany	29271730
C37	2018	Kim, Rapoport	To discuss the value of intraoperative CT and commercial freehand image guidance for cochlear implant surgery in patients with abnormal temporal bone anatomy	Usefulness for identification of anatomy	FIG	CI	NA	2	NA	NA	CT	0.62	Kolibri 2.0, Brainlab	Polaris, NDI	Optical	Freehand by surgeon	Anatomical landmarks	Utility of intraoperative computed tomography for cochlear implantation in patients with difficult anatomy	Albany, USA	29188758
C38	2019	Caversaccio, Weber	To assess the feasibility of cochlear keyhole access creation with a custom task-specific image guided robot and the associated keyhole approach for cochlear implantation	Number of completed access tunnels to the middle ear; Number of procedures with successful insertions and preservation of facial and chorda tympani nerve; Drilling error; Occurred extra- and intracochlear complications; Surgery time; Audiological outcomes	R	CI	0.48 (0.21 ± 0.09)	9	2D positioning error	Virtual target and trajectory definition in navigation image, automatic trajectory drilling to target, post-op imaging and coregistration to navigation image, manual drill hole segmentation, automatic deviation measurement between virtual trajectory and drill hole axis at target depth	CT	0.2	Custom, Custom	CamBar B1, Axios3D	Optical	Robotic manipulator	Bone screws	Robotic middle ear access for cochlear implantation: First in man	Bern, Switzerland	31374092
**Pre-clinical application (ex-vivo)**																				
E1	1997	Vrionis, Shea	To assess the feasibility and accuracy of anatomical landmark based registration for temporal bone surgery	Error; Usefulness for identification of anatomy	FIG	LSB	1.95 (1.05 ± 0.3)	10	Unknown	Anatomical target landmark, visual/manual identification of true position in image, manual digitization in patient with tracked instrument, manual/automatic deviation measurement between true and navigation-indicated position	CT	1	StealthStation, Medtronic	Polaris, NDI	Optical	Freehand by surgeon	Anatomical landmarks	Use of cranial surface anatomic fiducials for interactive image-guided navigation in the temporal bone: a cadaveric study.	Memphis, USA	9092849
E2	1997	Vrionis, Shea	To assess the value of commercial image guidance for the creation of a minimally invasive access to the internal auditory canal	Error; Usefulness for identification of anatomy	FIG	IAC	2.85 (1.28 ± 0.52)	9	Unknown	Anatomical target landmark, visual/manual identification of true position in image, manual digitization in patient with tracked instrument, manual/automatic deviation measurement between true and navigation-indicated position	CT	1	StealthStation, Medtronic	Polaris, NDI	Optical	Freehand by surgeon	Anatomical landmarks	Image-Interactive Orientation in the Middle Cranial Fossa Approach to the Internal Auditory Canal: An Experimental Study	Memphis, USA	9092849
E3	1998	Brinker, Samii	To assess the usefulness, reliability and accuracy of a commercial image guided microscope in lateral skull base surgery	Error; System operability; Usefulness for identification of anatomy	FIG	IAC	1.54 (0.69 ± 0.28)	10	3D positioning error	NA	CT	1	MKM, Carl Zeiss	NA	Mechanical	Freehand by surgeon	Bone screws	An experimental approach to image guided skull base surgery employing a microscope-based neuronavigation system.	Hannover, Germany	9842424
E4	2004	Schipper, Laszig	To assess the value of commercial freehand image guidance for cochlear implant surgery	Error; Controllability of surgical steps; Usefulness for identification of anatomy; /*Safety of surgery, */System operability (Practicability of anatomical landmark and screw fiducial registration)	FIG	CI	1.68 (1.2 ± 0.16)	1	3D positioning error	Anatomical target landmark, visual/manual identification of true position in image, manual digitization in patient with tracked instrument, manual/automatic deviation measurement between true and navigation-indicated position	CT	1	NA	Stryker, Stryker	Optical	Freehand by surgeon	Bone screws	Navigation as quality managment tool in cochlear implant surgery	Freiburg, Germany	15550181
E5	2005	Copeland, Pillsbury	To assess the accuracy of commercial freehand image guidance in neurotologic surgery	Error	FIG	LSB	1.19 (0.35 ± 0.28)	9	2D positioning error	Anatomical target landmark, visual/manual identification of true position in image, manual digitization in patient with tracked instrument, manual/automatic deviation measurement between true and navigation-indicated position	CT	0.75	Vectorvision, Brainlab	Polaris, NDI	Optical	Freehand by surgeon	Skin affixed landmarks	The accuracy of computer-aided surgery in neurotologic approaches to the temporal bone: a cadaver study	Chapel Hill, USA	15746856
E6	2007	Ledderose, Hagedorn	To assess the accuracy of commercial freehand image guidance and surface matching registration in lateral skull base surgery	Error	FIG	LSB	2.72 (1.13 ± 0.53)	2	3D positioning error	NA	CT	1	Vectorvision, Brainlab	Polaris, NDI	Optical	Freehand by surgeon	Skin surface matching	Surface laser registration in ENT-surgery: accuracy in the paranasal sinuses-a cadaveric study	Munich, Germany	18085021
E7	2007	Warren, Labadie	To present and assess the feasibility of custom microstereotactic frames for cochlear keyhole access creation	Number of access drillings to the middle ear without collision with structures to be preserved; Distances of drill to structures to be preserved; Occurred complications	SF	CI	NA	8	NA	NA	CT	0.8	Custom, Custom	NA	None	Microstereotactic frame	Bone screws	Percutaneous cochlear access using bone-mounted, customized drill guides: demonstration of concept in vitro	Nashville, USA	17414037
E8	2008	Majdani, Lenarz	To assess the feasibility of cochlear keyhole access and cochleostomy creation with commercial freehand image guidance	Topographical-anatomical description of created access tunnel; Occurred complications; System associated time expenditure	FIG	CI	NA	4	NA	NA	CT	0.15	Vectorvision, Brainlab	Polaris, NDI	Optical	Freehand by surgeon	Bone screws	A True Minimally Invasive Approach for Cochlear Implantation: High Accuracy in Cranial Base Navigation Through Flat-Panel-Based Volume Computed Tomography	Hannover, Germany	17971720
E9	2008	Pillai, Ammirati	To assess the accuracy of commercial freehand image guidance in neurotologic surgery	Error	FIG	LSB	1.75 (0.91 ± 0.28)	10	3D positioning error	Anatomical target landmark, visual/manual identification of true position in image, manual digitization in patient with tracked instrument, manual/automatic deviation measurement between true and navigation-indicated position	CT	0.6	NA	Stryker, Stryker	Optical	Freehand by surgeon	Bone screws	Application Accuracy of Computed Tomography-based, Image-guided Navigation of Temporal Bone	Columbus, USA	25908476
E10	2008	Stolka, Federspil	To assess the feasibility of implant bed milling in lateral skull base surgery with a custom ultrasound guided robot	Geometric error of milled implant bed shape; Number of occurred skull penetrations	R	CI	NA	5	NA	NA	Ultrasound	NA	Custom, Custom	Polaris, NDI	Optical	Robotic manipulator	Calibrated imaging device	First 3D ultrasound scanning, planning, and execution of CT-free milling interventions with a surgical robot	Bayreuth, Germany	19163988
E11	2009	Majdani, Leinung	To assess the feasibility of cochlear keyhole access and cochleostomy creation with an industrial robot adapted for image guidance, and to assess the feasibility of cochlear implantation through the associated keyhole approach	Drilling error; Number of completed access tunnels to the middle ear; Number of completed cochleostomies; Occurred complications; Distances of drill hole to structures to be preserved; Drill path length	R	CI	1.65 (0.78 ± 0.29)	10	2D positioning error	Virtual target and trajectory definition in navigation image, automatic trajectory drilling to target, post-op imaging and coregistration to navigation image, manual drill hole segmentation, automatic deviation measurement between virtual trajectory and drill hole axis at target depth	CT	0.13	Custom, Custom	Polaris, NDI	Optical	Robotic manipulator	Bone screws	A robot-guided minimally invasive approach for cochlear implant surgery: preliminary results of a temporal bone study	Hannover, Germany	20033531
E12	2009	Pillai, Ammirati	To assess the value of commercial freehand image guidance and endoscopy for creating a retrosigmoid access to the internal auditory canal	Error; Distances between anatomical structures; /*Safety of image guided approach (Usefulness to avoid iatrogenic injury)*/	FIG	IAC	0.94 (0.46 ± 0.16)	10	Unknown	NA	CT	0.6	NA	Stryker, Stryker	Optical	Freehand by surgeon	Bone screws	Image-guided, endoscopic-assisted drilling and exposure of the whole length of the internal auditory canal and its fundus with preservation of the integrity of the labyrinth using a retrosigmoid approach: a laboratory investigation	Columbus, USA	19935002
E13	2010	Balachandran, Labadie	To assess the feasibility of cochlear keyhole access creation with custom microstereotactic frames	Number of completed access tunnels to the middle ear; Number of access tunnels without contact between the drill and the facial nerve; Occurred complications; Distances of drill hole axis to structures to be preserved; Drill time; Error	SF	CI	0.61 (0.31 ± 0.1)	6	2D positioning error	Virtual target and trajectory definition in navigation image, automatic trajectory drilling to target, drill into drill hole, post-op imaging and coregistration to navigation image, automatic drill segmentation, automatic deviation measurement between virtual trajectory and drill axis at target depth	CT	0.4	Custom, Custom	NA	None	Microstereotactic frame	Bone screws	Percutaneous cochlear implant drilling via customized frames: an in vitro study	Nashville, USA	20172392
E14	2011	Dalgorf, Irish	To assess the accuracy of automatic calibrated CT based registration for lateral skull base surgery	Error	FIG	LSB	1.66 (0.91 ± 0.25)	1	Unknown	NA	CT	NA	Custom, Custom	NA	NA	Freehand by surgeon	Calibrated imaging device	Accuracy and reproducibility of automatic versus manual registration using a cone-beam CT image guidance system	Toronto, Canada	21303606
E15	2011	Kral, Freysinger	To assess the accuracy of image guidance for lateral skull base surgery for different registration methods and image resolutions	Error	FIG	LSB	1.56 (0.72 ± 0.28)	1	3D positioning error	NA	CT	0.5	STN, Carl Zeiss	FlashPoint 3000, Image Guided Technologies	Optical	Freehand by surgeon	Hybrid	Navigated surgery at the lateral skull base and registration and preoperative imagery: experimental results	Innsbruck, Austria	21339400
E16	2011	Kratchman, Webster	To present and assess the feasibility of a custom robotic microstereotactic frame for the creation of cochlear keyhole access	Drilling error; System operability	RSF	CI	($\mu = 0.38$)	1	3D positioning error	Virtual target and trajectory definition in navigation image, automatic trajectory drilling to target, drill into drill hole, post-op imaging and coregistration to navigation image, automatic drill segmentation, automatic deviation measurement between virtual trajectory and drill axis at target depth	CT	0.4	Custom, Custom	NA	None	Robotic microstereotactic frame	Bone screws	Design of a Bone-Attached Parallel Robot for Percutaneous Cochlear Implantation Â	Nashville, USA	21788181
E17	2011	Nguyen, Grayeli	To assess the feasibility of cochlear keyhole access and cochleostomy creation with a commercial freehand image guidance system, and to assess the feasibility of cochlear implantation through the associated keyhole approach	Error range; Number of created tunnels enabling access to the round window; Number of geometrically correct cochleostomy locations; Number of insertions with the electrode penetrating into the cochlea at the level of the scala tympani; Number of drillings without injury to structures to be preserved; Occurred complications; Keyhole access position and orientation relative to ear canal and temporal bone squama; Access tunnel length	FIG	CI	NA	5	NA	NA	CT	0.3	Digipointeur, Collin	Collin, Collin	Electromagnetic	Freehand by surgeon	Bone screws	Minimally invasive computer-assisted approach for cochlear implantation: a human temporal bone study	Paris, France	21502203
E18	2011	Stieger, Weber	To present and assess the feasibility of a custom image guided robot for cochlear keyhole access creation	Work field dimensions; Drilling error	R	CI	($\mu = 3.42$)	1	2D positioning error	Virtual target and trajectory definition in navigation image, automatic trajectory drilling to target, post-op imaging and coregistration to navigation image, manual drill hole segmentation, automatic deviation measurement between virtual trajectory and drill hole axis at target depth	CT	0.62	Custom, Custom	Polaris, NDI	Optical	Robotic manipulator	Skin surface matching	Development of an auditory implant manipulator for minimally invasive surgical insertion of implantable hearing devices	Bern, Switzerland	21078217
E19	2012	Bell, Weber	To present and assess the accuracy of a custom task-specific image guided robot for cochlear implant surgery	Error; Number of completed middle ear access tunnels; Occurred complications; System associated time	R	CI	1.79 (0.56 ± 0.41)	15	2D positioning error	Virtual target and trajectory definition in navigation image, automatic trajectory drilling to target, titanium rod into drill hole, post-op imaging and coregistration to navigation image, automatic rod segmentation, automatic deviation measurement between virtual trajectory and rod axis at target depth	CT	0.15	Custom, Custom	CamBar B1, Axios3D	Optical	Robotic manipulator	Bone screws	A self-developed and constructed robot for minimally invasive cochlear implantation.	Bern, Switzerland	22385333
E20	2012	Kohan, Jethanamest	To assess the value of commercial freehand image guidance in otologic surgery	System associated time; Number of occurred complications; Error; System operability	FIG	LSB	1.34 (0.92 ± 0.14)	15	Unknown	Anatomical target landmark, visual/manual identification of true position in image, manual digitization in patient with tracked instrument, manual/automatic deviation measurement between true and navigation-indicated position	CT	1	LandmarX, Medtronic	Polaris, NDI	Optical	Freehand by surgeon	Skin affixed landmarks	Image-Guided Surgical Navigation in Otology	New York, USA	22961537
E21	2012	Kratchman, Labadie	To assess the feasibility of cochlear implant insertion with a custom manually operated image guided insertion device	Number of full insertions; Occurred intracochlear complications	SF	CI	NA	3	NA	NA	CT	NA	Custom, Custom	NA	None	Microstereotactic frame	Bone screws	A Manually Operated, Advance Off-Stylet Insertion Tool for Minimally Invasive Cochlear Implantation Surgery	Nashville, USA	22851233
E22	2013	Bell, Weber	To assess the drilling accuracy of a custom task-specific image guided robot for cochlear implantation, to assess the feasibility of keyhole access creation with the robot and to assess the feasibility of the associated keyhole approach for cochlear implantation	Drilling error; System associated time; Number of full/partial insertions	R	CI	0.39 (0.15 ± 0.08)	8	2D positioning error	Virtual target and trajectory definition in navigation image, automatic trajectory drilling to target, titanium rod into drill hole, post-op imaging and coregistration to navigation image, automatic rod segmentation, automatic deviation measurement between virtual trajectory and rod axis at target depth	CT	0.2	Custom, Custom	CamBar B1, Axios3D	Optical	Robotic manipulator	Bone screws	In vitro accuracy evaluation of image-guided robot system for direct cochlear access	Bern, Switzerland	23921934
E23	2013	Gerber, Weber	To assess the accuracy of refined bone screw registration (automatic identification and digitization in the image and on the patient respectively) and custom image guidance for cochlear implant surgery	Error	FIG	CI	0.13 (0.07 ± 0.02)	1	3D positioning error	Artificial target fiducial, automatic identification of true position in image, manual digitization in patient with tracked instrument, automatic deviation measurement between true and navigation-indicated position	CT	0.15	Custom, Custom	CamBar B1, Axios3D	Optical	Freehand by surgeon	Bone screws	High-Accuracy Patient-to-Image Registration for the Facilitation of Image-Guided Robotic Microsurgery on the Head	Bern, Switzerland	23340586
E24	2013	Kral, Freysinger	To compare the accuracy of optical and electromagnetic commercial freehand image guidance for lateral skull base surgery	Error	FIG	LSB	1.38 (0.22 ± 0.38)	6	3D positioning error	NA	CT	1	StealthStation, Medtronic	Polaris, NDI	Optical	Freehand by surgeon	Skin affixed landmarks	Comparison of optical and electromagnetic tracking for navigated lateral skull base surgery	Innsbruck, Austria	23661534
E25	2013	McRackan, Labadie	To assess the feasibility of implanting electrodes from different manufactures through a keyhole access to the cochlea created with custom microstereotactic frames	Number of successful insertions; Insertion depth (degrees, full/partial); Occurred extra- and intracochlear complications	SF	CI	NA	7	NA	NA	CT	0.4	Custom, Custom	NA	None	Microstereotactic frame	Bone screws	Validation of Minimally-Invasive, Image-guided Cochlear Implantation Using Advanced Bionics, Cochlear, and Medel Electrodes in a Cadaver Model	Nashville, USA	23633113
E26	2014	Kral, Freysinger	To assess the accuracy of freehand image guidance and a custom patient tracker for lateral skull base surgery	Error	FIG	LSB	4.72 (2.08 ± 0.88)	1	3D positioning error	NA	CT	0.6	Custom, Custom	NA	Electromagnetic	Freehand by surgeon	Bone screws	A new nasopharyngeal dynamic reference frame improves accuracy in navigated skull base targets	Innsbruck, Austria	24108364
E27	2014	Nau-Hermes, Schipper	To present and assess the value of quality gates for multiport image guided robotic surgery	Quality gates; Effectiveness of quality gates for assuring safe surgery	R	CI	NA	1	NA	NA	NA	NA	Custom, Custom	NA	None	Robotic manipulator	Skin affixed landmarks	Quality Assurance of Multiport Image-Guided Minimally Invasive Surgery at the Lateral Skull Base	Aachen, Germany	25105146
E28	2014	Rohani, Labadie	To assess forces in the cochlea during cochlear implant insertion through a keyhole cochlear access	Insertion forces; Insertion depth (degrees, number of intracochlear electrode contacts); Occurred intracochlear complications	SF	CI	NA	6	NA	NA	CT	NA	Custom, Custom	NA	None	Microstereotactic frame	Bone screws	Forces and trauma associated with minimally invasive image-guided cochlear implantation	Nashville, USA	24468898
E29	2015	Afifi, Grayeli	To assess the value of commercial freehand image guidance for, and the feasibility of an image guided transmodiolar approach to the internal auditory canal	Error; Feasibility of image guided surgical approach	FIG	IAC	0.24 (0.06 ± 0.06)	6	3D positioning error	Anatomical target landmark, visual/manual identification of true position in image, manual digitization in patient with tracked instrument, manual/automatic deviation measurement between true and navigation-indicated position	CT	0.3	Digipointeur, Collin	Collin, Collin	Electromagnetic	Freehand by surgeon	Bone screws	Navigation-guided transmodiolar approach for auditory nerve implantation via the middle ear in humans	Dijon, France	25791165
E30	2015	Colasanti, Ammirati	To assess the value of commercial freehand image guidance for identifying the endolymphatic sac and vestibular aqueduct during retrosigmoid approaches	Usefulness for identification of anatomy; Distances between anatomical structures	FIG	LSB	NA	12	NA	NA	CT	0.6	NA	Stryker, Stryker	Optical	Freehand by surgeon	Bone screws	Image-guided, microsurgical topographic anatomy of the endolymphatic sac and vestibular aqueduct via a suboccipital retrosigmoid approach	Columbus, USA	25908476
E31	2015	Colasanti, Ammirati	To describe and assess the feasibility of an image guided (commercial freehand system) retrosigmoid access for exposing the intrapetrous carotid artery	Topographical-anatomical description of access route (Distances between anatomical structures); Adequate exposure of intrapetrous carotid artery achieved (Y/N); Occurred complications; Usefulness of image guidance for identification of anatomy and extent of access tunnel	FIG	LSB	NA	12	NA	NA	CT	0.6	NA	Stryker, Stryker	Optical	Freehand by surgeon	Bone screws	Maximizing the petroclival region exposure via a suboccipital retrosigmoid approach: where is the intrapetrous internal carotid artery?	Columbus, USA	25830604
E32	2016	Ke, Ma	To assess the accuracy of freehand image guidance and different registration methods for cochlear implant surgery	Error	FIG	CI	1.39 (0.64 ± 0.25)	10	3D positioning error	NA	CT	0.6	Custom, Custom	Polaris, NDI	Optical	Freehand by surgeon	Bone screws	Application of bonebed-malleus short process registration in minimally invasive cochlear implantation	Beijing, China	NA
E33	2016	Kempfle, Lee	To assess the value of commercial freehand image guidance for, and the feasibility of an image guided endoscopic cochlea-preserving transcanal approach for internal auditory canal surgery	Error; Usefulness for identification of anatomy; Number of occurred complications	FIG	IAC	0.7 (0.4 ± 0.1)	3	Unknown	NA	CT	NA	Fusion, Medtronic	NA	Electromagnetic	Freehand by surgeon	Bone screws	Endoscopic Transcanal Retrocochlear Approach to the Internal Auditory Canal with Cochlear Preservation: Pilot Cadaveric Study	Boston, USA	26932951
E34	2016	Komune, Rhoton	To assess the accuracy of commercially and clinically available electromagnetic image guidance	Error; Usefulness for avoiding iatrogenic injury; Description of successfully completed procedures	FIG	LSB	0.64 (0.49 ± 0.05)	20	3D positioning error	Anatomical target landmark, visual/manual identification of true position in image, manual digitization in patient with tracked instrument, manual/automatic deviation measurement between true and navigation-indicated position	NA	0.6	StealthStation, Medtronic	NA	Electromagnetic	Freehand by surgeon	Bone screws	The Accuracy of an Electromagnetic Navigation System in Lateral Skull Base Approaches	Fukuoka, Japan	27301466
E35	2016	Zhou, Williamson	To assess the accuracy of a custom freehand image guidance system and surface matching registration for lateral skull base surgery	Error	FIG	LSB	0.49 (0.2 ± 0.1)	14	3D positioning error	Artificial target fiducial, automatic identification of true position in image, manual digitization in patient with tracked instrument, automatic deviation measurement between true and navigation-indicated position	CT	0.2	Custom, Custom	CamBar B1, Axios3D	Optical	Freehand by surgeon	Bone surface matching	Surface matching for high-accuracy registration of the lateral skull base	Bern, Switzerland	27142458
E36	2017	Dillon, Labadie	To assess the feasibility of a custom microstereotactic frame mounted robot for access creation to the internal auditory canal	Milled bone volume; Error; Milling time; Number of procedures without injury facial nerve or chorda tympani	RSF	IAC	NA	5	NA	NA	CT	0.4	Custom, Custom	NA	None	Robotic microstereotactic frame	Bone screws	Cadaveric Testing of Robot-Assisted Access to the Internal Auditory Canal for Vestibular Schwannoma Removal	Nashville, USA	28079677
E37	2017	Ding, Qian	To assess the value of commercial freehand image guidance for, and the feasibility of a endoscopic image guided retrosigmoid approach	Topographical-anatomical description of approach; Exposure of relevant anatomy and area of exposure; Thickness of milled bone; Error; Number of occurred complications image guided vs. non-image guided	FIG	LSB	0.75 (0.48 ± 0.09)	20	Unknown	NA	CT	1	MATRIX POLAR, XION	Polaris, NDI	Optical	Freehand by surgeon	NA	Endoscopic Intradural Subtemporal Keyhole Approach with Neuronavigational Assistance to the Suprasellar, Petroclival, and Ventrolateral Brainstem Regions: An Anatomic Study	Wuxi, China	28223248
E38	2017	Matsushima, Kohno	To assess the value of commercial freehand image guidance for, and the feasibility of image guided extensions of the retrosigmoid approach	Topographical-anatomical description of approach; Number of occurred complications; Error	FIG	LSB	1.03 (0.43 ± 0.2)	20	3D positioning error	Anatomical target landmark, visual/manual identification of true position in image, manual digitization in patient with tracked instrument, manual/automatic deviation measurement between true and navigation-indicated position	CT	0.63	StealthStation, Medtronic	NA	Electromagnetic	Freehand by surgeon	Bone screws	Microsurgical and Endoscopic Anatomy for Intradural Temporal Bone Drilling and Applications of the Electromagnetic Navigation System: Various Extensions of the Retrosigmoid Approach	Tokyo, Japan	28435113
E39	2017	Torres, Nguyen	To compare insertion angles achieved using a commercial freehand image guidance system and an image guided robot	Insertion angles; System associated time	FIG	CI	NA	4	NA	NA	CT	0.12	Digipointeur, Collin	Collin, Collin	Electromagnetic	Freehand by surgeon	Anatomical landmarks	Improvement of the insertion axis for cochlear implantation with a robot-based system	Paris, France	27704279
E40	2017	Weber, Caversaccio	To present an image guided robotic treatment model for cochlear implant surgery, its application to custom image guidance technology, and its application in a first patient	Description of robotic treatment model for cochlear implantation and its implementation with custom image guidance technology; Feasibility in a first patient (Feasibility of middle ear access creation and implant insertion; Insertion depth (degrees, number of intracochlear electrode contacts); Audiological outcomes; Occurred complications)	R	CI	NA	NA	NA	NA	CT	0.2	Custom, Custom	CamBar B1, Axios3D	Optical	Robotic manipulator	Bone screws	Instrument flight to the inner ear	Bern, Switzerland	30246168
E41	2018	McJunkin, Silva	To assess the accuracy of a custom image guided mixed reality system for lateral skull base surgery	System operability; Error	FIG	LSB	7.38 (5.76 ± 0.54)	1	3D positioning error	Anatomical target landmark, visual/manual identification of true position in image, manual digitization in patient with tracked instrument, manual/automatic deviation measurement between true and navigation-indicated position	CT	0.6	Custom, Custom	Optotrak, NDI	Optical	Freehand by surgeon	Skin surface matching	Development of a Mixed Reality Platform for Lateral Skull Base Anatomy	St. Louis, USA	30239435
E42	2018	Rathgeb, Williamson	To assess the accuracy and usability of a custom freehand image guidance system for lateral skull base surgery	Error; Usefulness for identification of anatomy; System operability (Operating room, surgical site and surgical workflow integration)	FIG	LSB	0.58 (0.28 ± 0.1)	3	3D positioning error	Artificial target fiducial, automatic identification of true position in image, manual digitization in patient with tracked instrument, automatic deviation measurement between true and navigation-indicated position	CT	0.2	Custom, Custom	CamBar B1, Axios3D	Optical	Freehand by surgeon	Bone screws	Accuracy and feasibility of a dedicated image guidance solution for endoscopic lateral skull base surgery	Bern, Switzerland	29442163
E43	2018	Schneider, Anschuetz	To assess the accuracy of a custom freehand image guidance and anatomy based registration methods for lateral skull base surgery	Error	FIG	LSB	0.75 (0.36 ± 0.13)	2	3D positioning error	Artificial target fiducial, automatic identification of true position in image, manual digitization in patient with tracked instrument, automatic deviation measurement between true and navigation-indicated position	CT	0.2	Custom, Custom	CamBar B1, Axios3D	Optical	Freehand by surgeon	Bone surface matching	Noninvasive Registration Strategies and Advanced Image Guidance Technology for Submillimeter Surgical Navigation Accuracy in the Lateral Skull Base	Bern, Switzerland	30239434
E44	2019	Schneider, Klenzner	To assess the feasibility of cochlear implant electrode insertion through a multiport approach created with a commercial task-specific image guided robot	Number of successful electrode insertions; Video of all insertions; Drilling error; Number of plannable trajectories per access route; Number of completed access tunnels to the middle ear; Number of completed inner ear accesses without damaging the round window membrane; Insertion angles; Insertion depth (degrees, number of intracochlear electrode contacts); Insertion time; Occurred complications	R	CI	0.4 (0.13 ± 0.09)	4	2D positioning error	Virtual target and trajectory definition in navigation image, automatic trajectory drilling to target, titanium rod into drill hole, post-op imaging and coregistration to navigation image, automatic rod segmentation, automatic deviation measurement between virtual trajectory and rod axis at target depth	CT	0.1	HEARO, CAScination	CamBar B1, Axios3D	Optical	Robotic manipulator	Bone screws	Robotic cochlear implantation: feasibility of a multiport approach in an ex vivo model	Bern, Switzerland	30739180
E45	2019	Schwam, Wanna	To assess the accuracy of commercial freehand image guidance for temporal bone surgery	Error	FIG	LSB	1.35 (0.48 ± 0.29)	18	3D positioning error	NA	CT	0.5	StealthStation, Medtronic	NA	Electromagnetic	Freehand by surgeon	Bone screws	Accuracy of a Modern Intraoperative Navigation System for Temporal Bone Surgery in a Cadaveric Model	New York, USA	31547788
