# Supplementary material for: Evolution and Stagnation of Image Guidance for Surgery in the Lateral Skull: A Systematic Review 1989–2020
Source: Front Surg. 2021 Jan 11;7:604362. doi: 10.3389/fsurg.2020.604362 (PMC7831154; doi:10.3389/fsurg.2020.604362)
Supplement: Supplementary file 2 [file Data_Sheet_2.PDF]

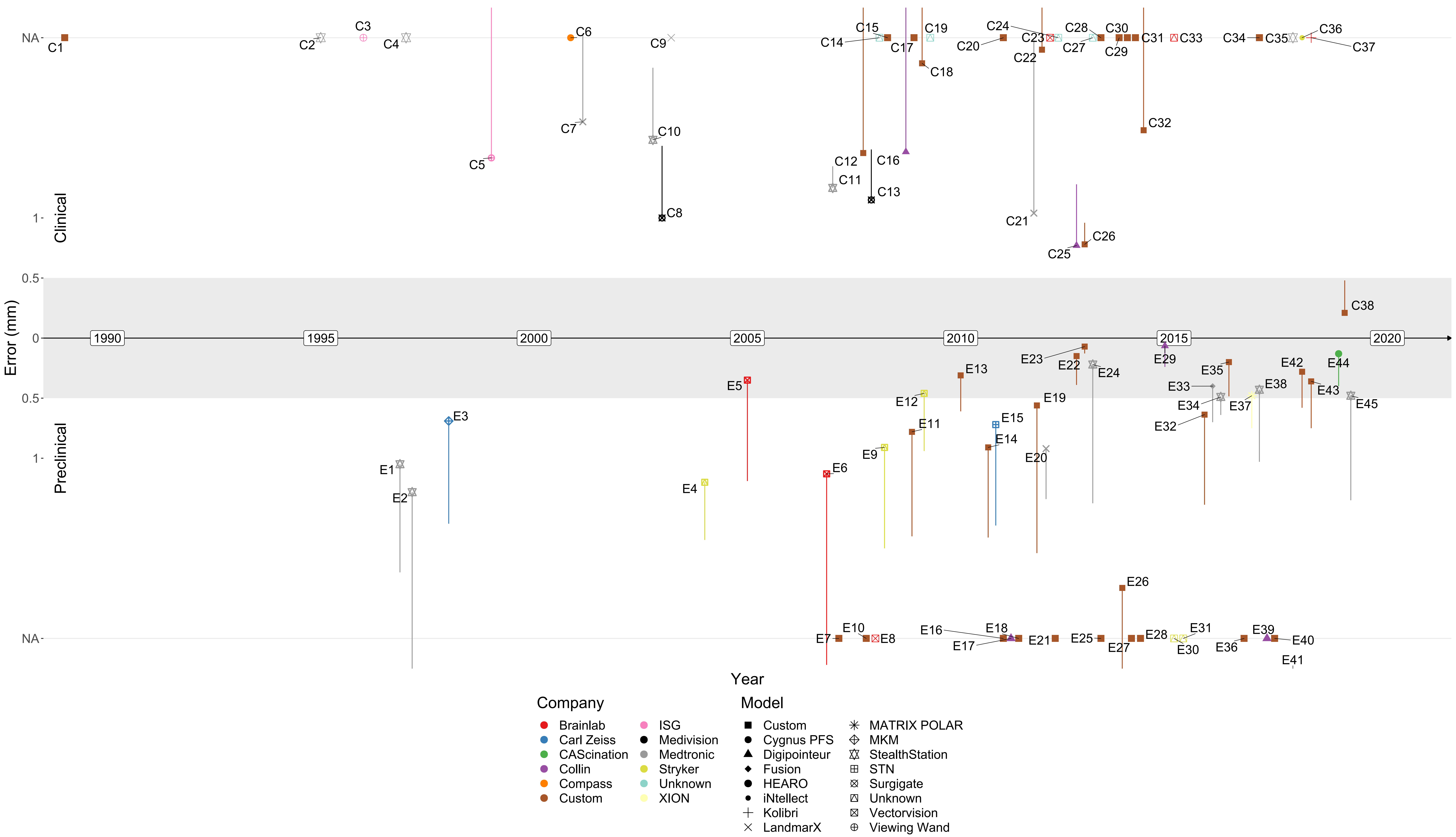

Clinical
Preclinical
Error (mm)
Year
NA
1
0.5
0
1990
1995
2000
2005
2010
2015
2020
Company
Brainlab
Carl Zeiss
CAScination
Collin
Compass
Custom
ISG
Medivision
Medtronic
Stryker
Unknown
XION
Model
Custom
Cygnus PFS
Digipointeur
Fusion
HEARO
iNtellect
Kolibri
LandmarX
MATRIX POLAR
MKM
StealthStation
STN
Surgigate
Unknown
Vectorvision
Viewing Wand

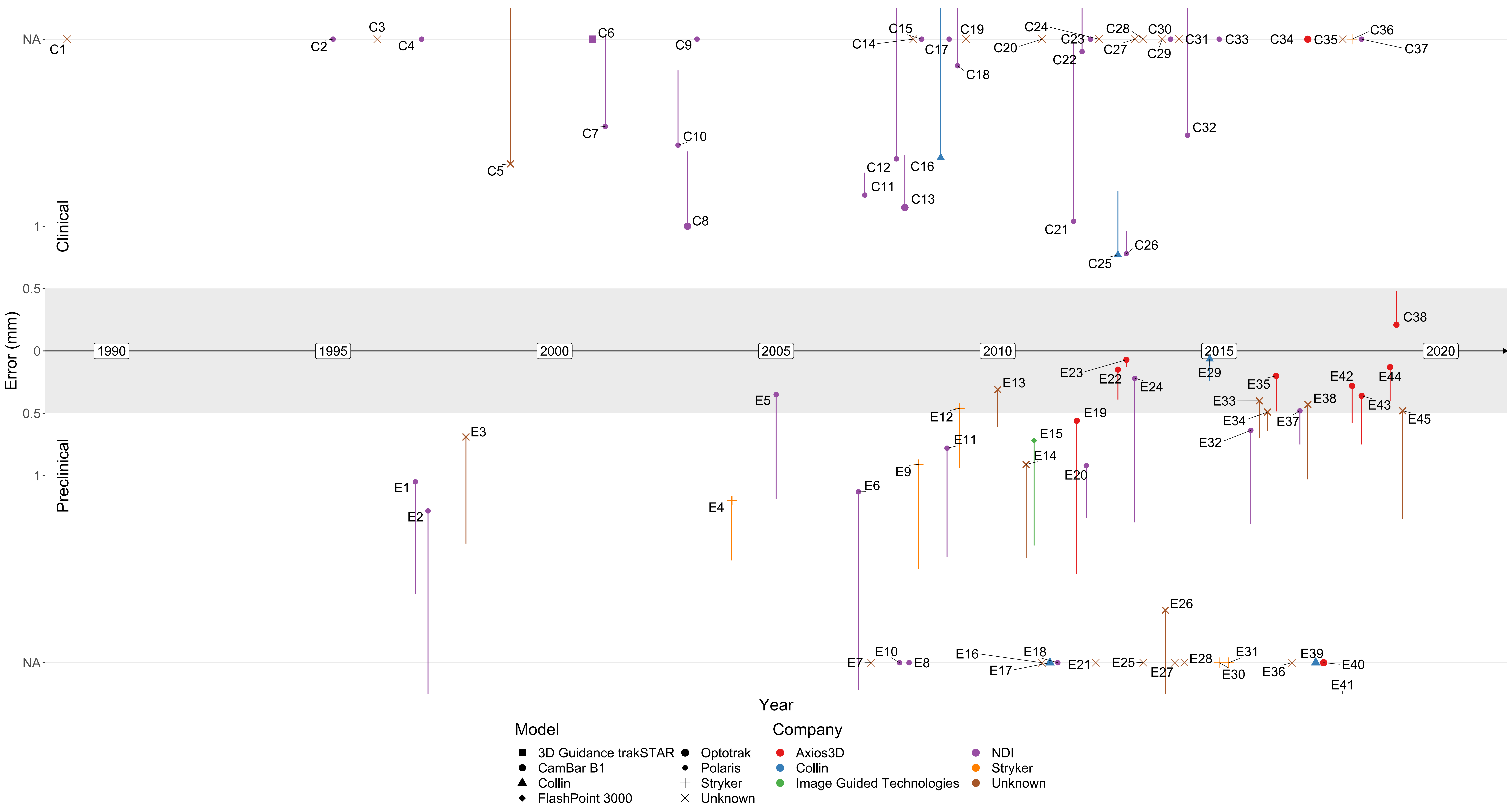

Clinical
Preclinical
Error (mm)
NA
1
0.5
0
0.5
1
NA
1990
1995
2000
2005
2010
2015
2020
Year
C1 C2 C3 C4 C5 C6 C7 C8 C9 C10 C11 C12 C13 C14 C15 C16 C17 C18 C19 C20 C21 C22 C23 C24 C25 C26 C27 C28 C29 C30 C31 C32 C33 C34 C35 C36 C37 C38
E1 E2 E3 E4 E5 E6 E7 E8 E9 E10 E11 E12 E13 E14 E15 E16 E17 E18 E19 E20 E21 E22 E23 E24 E25 E26 E27 E28 E29 E30 E31 E32 E33 E34 E35 E36 E37 E38 E39 E40 E41 E42 E43 E44 E45
Model
3D Guidance trakSTAR
CamBar B1
Collin
FlashPoint 3000
Optotrak
Polaris
Stryker
Unknown
Company
Axios3D
Collin
Image Guided Technologies
NDI
Stryker
Unknown

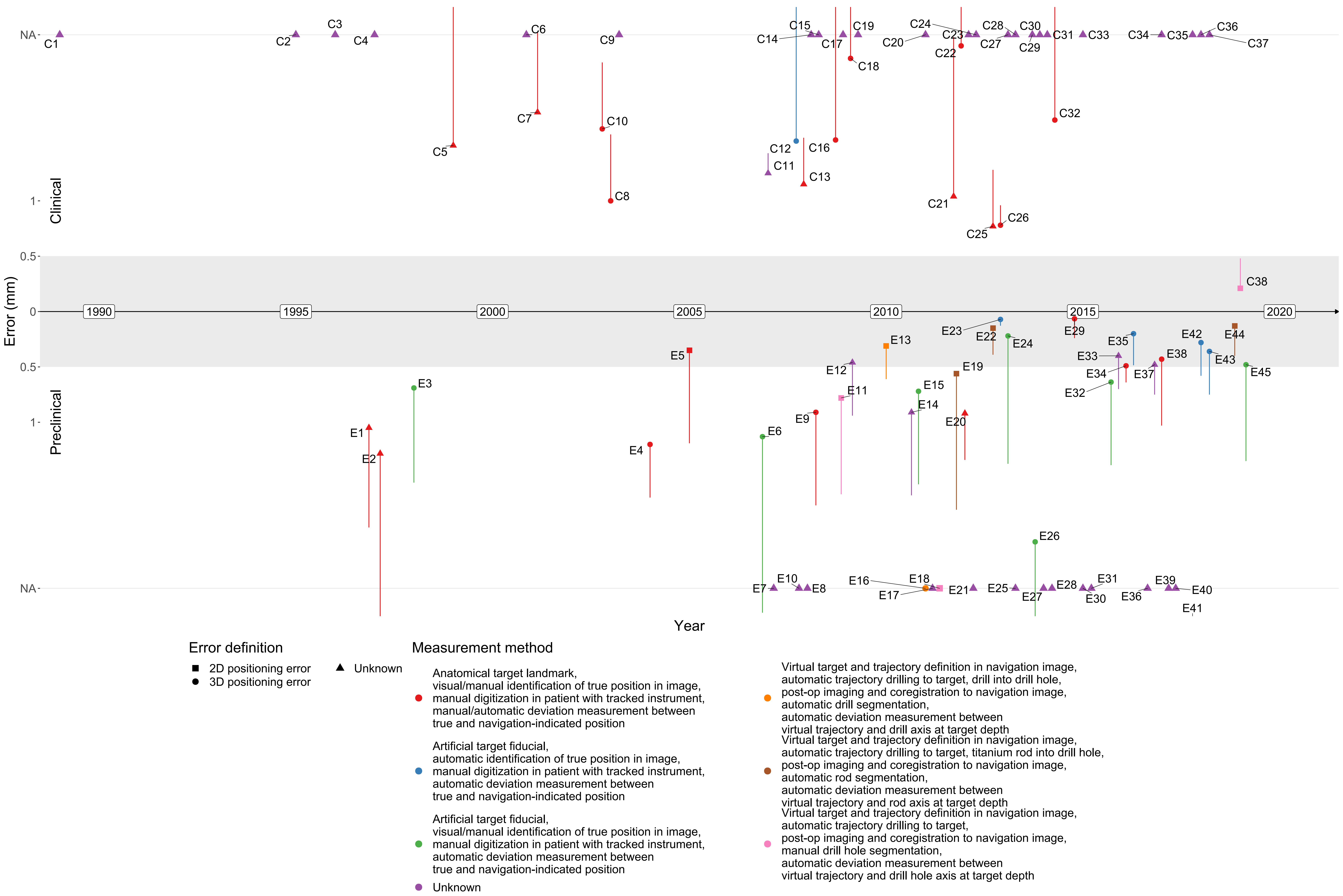

Clinical
Preclinical
Error (mm)
NA
1
0.5
0
Year
1990
1995
2000
2005
2010
2015
2020
C1 C2 C3 C4 C5 C6 C7 C8 C9 C10 C11 C12 C13 C14 C15 C16 C17 C18 C19 C20 C21 C22 C23 C24 C25 C26 C27 C28 C29 C30 C31 C32 C33 C34 C35 C36 C37 C38
E1 E2 E3 E4 E5 E6 E7 E8 E9 E10 E11 E12 E13 E14 E15 E16 E17 E18 E19 E20 E21 E22 E23 E24 E25 E26 E27 E28 E29 E30 E31 E32 E33 E34 E35 E36 E37 E38 E39 E40 E41 E42 E43 E44 E45
Error definition
2D positioning error
3D positioning error
Unknown
Measurement method
Anatomical target landmark, visual/manual identification of true position in image, manual digitization in patient with tracked instrument, manual/automatic deviation measurement between true and navigation-indicated position
Artificial target fiducial, automatic identification of true position in image, manual digitization in patient with tracked instrument, automatic deviation measurement between true and navigation-indicated position
Artificial target fiducial, visual/manual identification of true position in image, manual digitization in patient with tracked instrument, automatic deviation measurement between true and navigation-indicated position
Unknown
Virtual target and trajectory definition in navigation image, automatic trajectory drilling to target, drill into drill hole, post-op imaging and coregistration to navigation image, automatic drill segmentation, automatic deviation measurement between virtual trajectory and drill axis at target depth
Virtual target and trajectory definition in navigation image, automatic trajectory drilling to target, titanium rod into drill hole, post-op imaging and coregistration to navigation image, automatic rod segmentation, automatic deviation measurement between virtual trajectory and rod axis at target depth
Virtual target and trajectory definition in navigation image, automatic trajectory drilling to target, post-op imaging and coregistration to navigation image, manual drill hole segmentation, automatic deviation measurement between virtual trajectory and drill hole axis at target depth

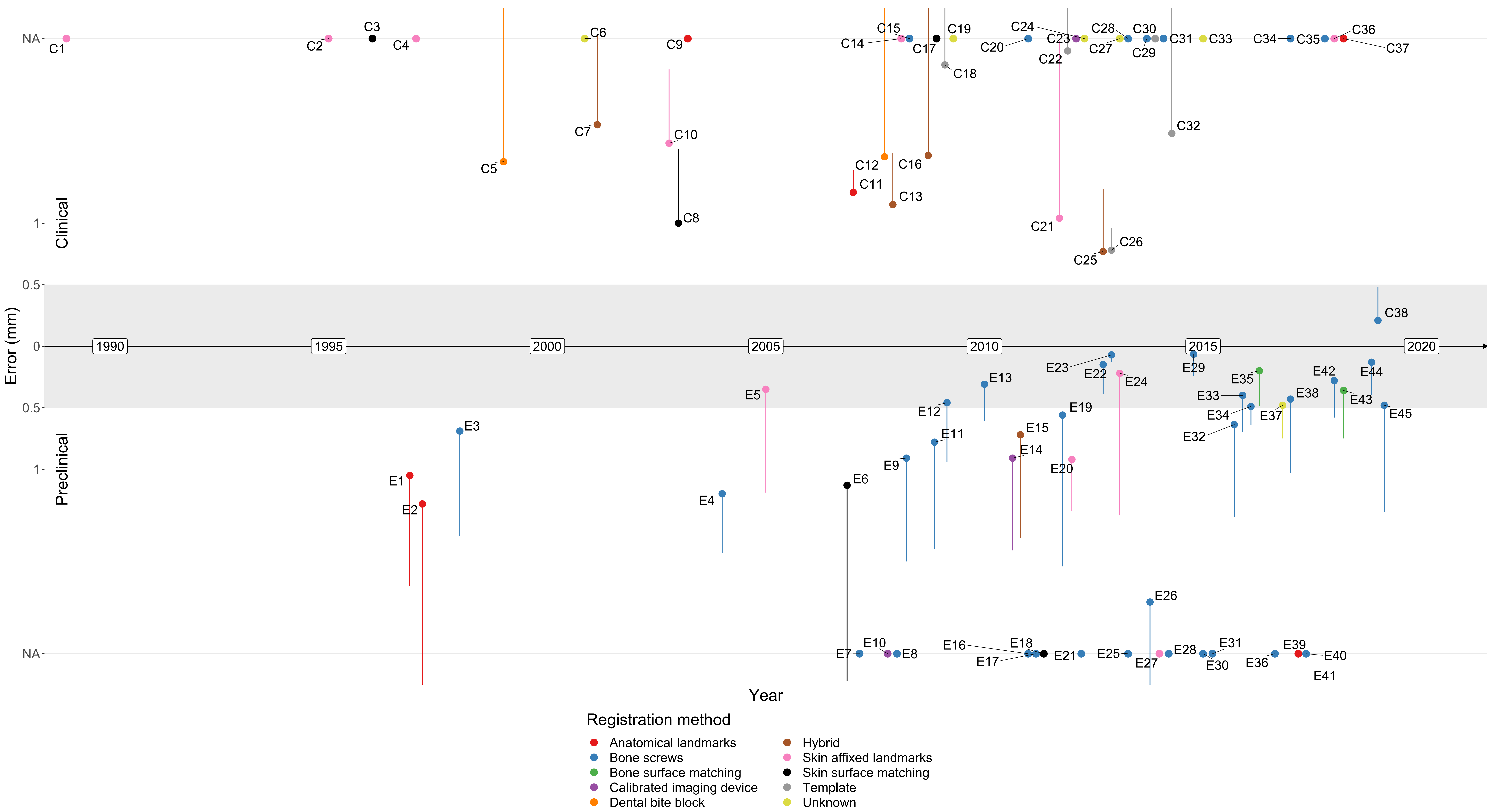

Clinical
Preclinical
Error (mm)
NA
1
0.5
0
1990
1995
2000
2005
2010
2015
2020
Year
C1 C2 C3 C4 C5 C6 C7 C8 C9 C10 C11 C12 C13 C14 C15 C16 C17 C18 C19 C20 C21 C22 C23 C24 C25 C26 C27 C28 C29 C30 C31 C32 C33 C34 C35 C36 C37 C38
E1 E2 E3 E4 E5 E6 E7 E8 E9 E10 E11 E12 E13 E14 E15 E16 E17 E18 E19 E20 E21 E22 E23 E24 E25 E26 E27 E28 E29 E30 E31 E32 E33 E34 E35 E36 E37 E38 E39 E40 E41 E42 E43 E44 E45
Registration method
Anatomical landmarks
Bone screws
Bone surface matching
Calibrated imaging device
Dental bite block
Hybrid
Skin affixed landmarks
Skin surface matching
Template
Unknown

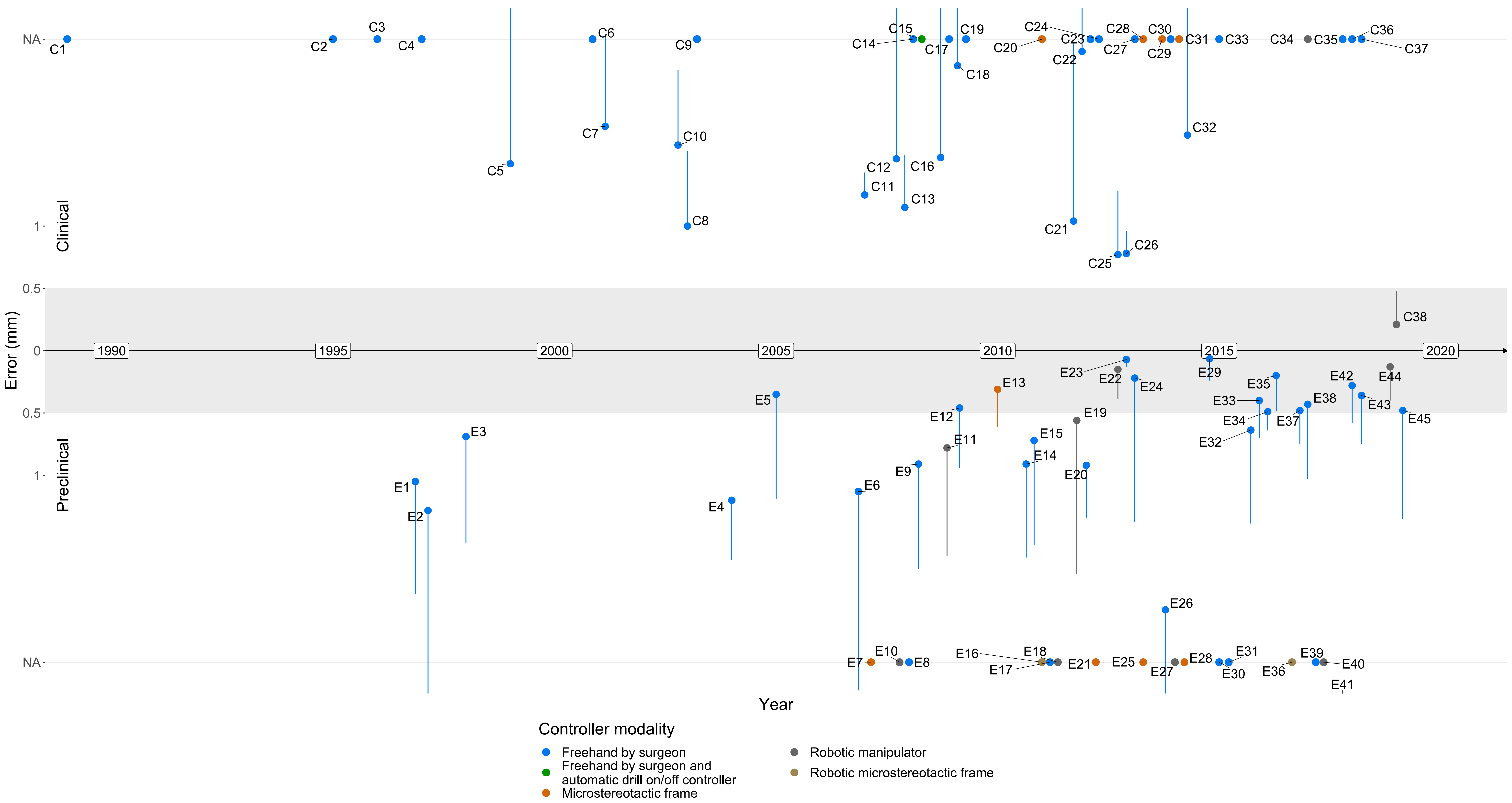

Clinical
Preclinical
Error (mm)
NA
1
0.5
0
0.5
1
NA
1990
1995
2000
2005
2010
2015
2020
Year
C1 C2 C3 C4 C5 C6 C7 C8 C9 C10 C11 C12 C13 C14 C15 C16 C17 C18 C19 C20 C21 C22 C23 C24 C25 C26 C27 C28 C29 C30 C31 C32 C33 C34 C35 C36 C37 C38
E1 E2 E3 E4 E5 E6 E7 E8 E9 E10 E11 E12 E13 E14 E15 E16 E17 E18 E19 E20 E21 E22 E23 E24 E25 E26 E27 E28 E29 E30 E31 E32 E33 E34 E35 E36 E37 E38 E39 E40 E41 E42 E43 E44 E45
Controller modality
Freehand by surgeon
Freehand by surgeon and automatic drill on/off controller
Microstereotactic frame
Robotic manipulator
Robotic microstereotactic frame

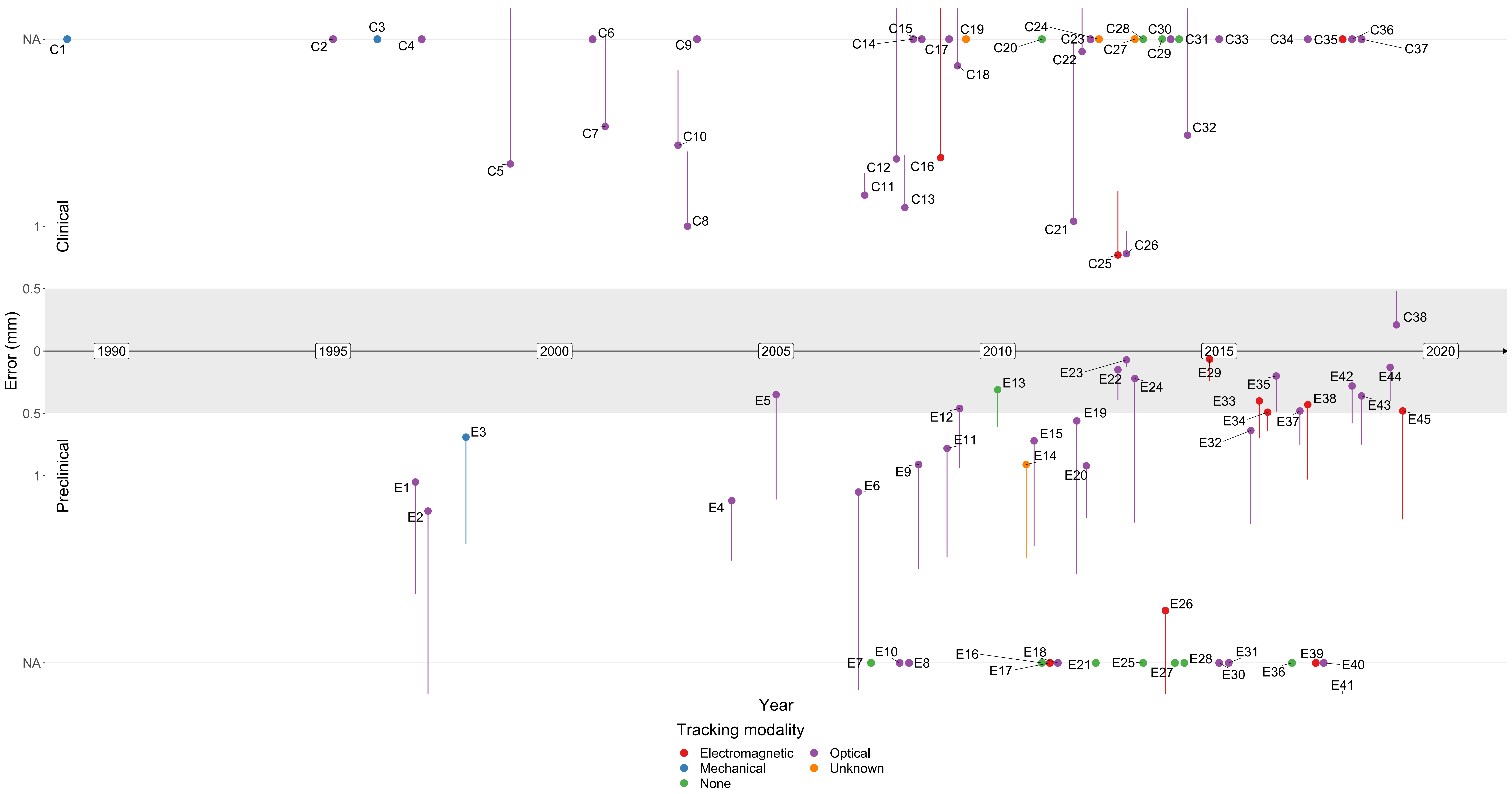
Error (mm)
Clinical
Preclinical
NA
1
0.5
0
0.5
1
NA
1990
1995
2000
2005
2010
2015
2020
C1 C2 C3 C4 C5 C6 C7 C8 C9 C10 C11 C12 C13 C14 C15 C16 C17 C18 C19 C20 C21 C22 C23 C24 C25 C26 C27 C28 C29 C30 C31 C32 C33 C34 C35 C36 C37 C38
E1 E2 E3 E4 E5 E6 E7 E8 E9 E10 E11 E12 E13 E14 E15 E16 E17 E18 E19 E20 E21 E22 E23 E24 E25 E26 E27 E28 E29 E30 E31 E32 E33 E34 E35 E36 E37 E38 E39 E40 E41 E42 E43 E44 E45
Year
Tracking modality
Electromagnetic
Mechanical
None
Optical
Unknown

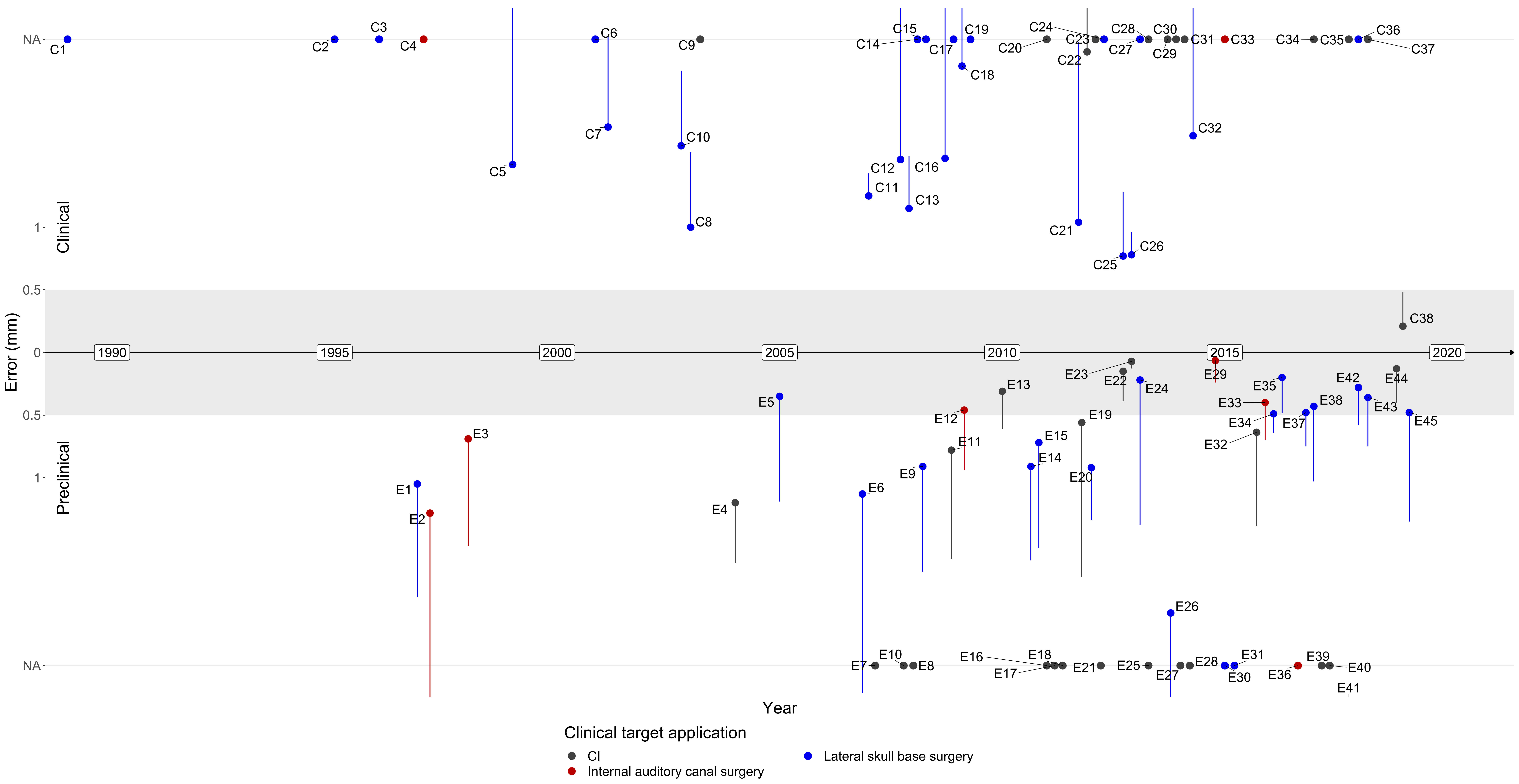

Clinical
Preclinical
Error (mm)
NA
1
0.5
0
1990
1995
2000
2005
2010
2015
2020
Year
C1 C2 C3 C4 C5 C6 C7 C8 C9 C10 C11 C12 C13 C14 C15 C16 C17 C18 C19 C20 C21 C22 C23 C24 C25 C26 C27 C28 C29 C30 C31 C32 C33 C34 C35 C36 C37 C38
E1 E2 E3 E4 E5 E6 E7 E8 E9 E10 E11 E12 E13 E14 E15 E16 E17 E18 E19 E20 E21 E22 E23 E24 E25 E26 E27 E28 E29 E30 E31 E32 E33 E34 E35 E36 E37 E38 E39 E40 E41 E42 E43 E44 E45
Clinical target application
CI
Internal auditory canal surgery
Lateral skull base surgery

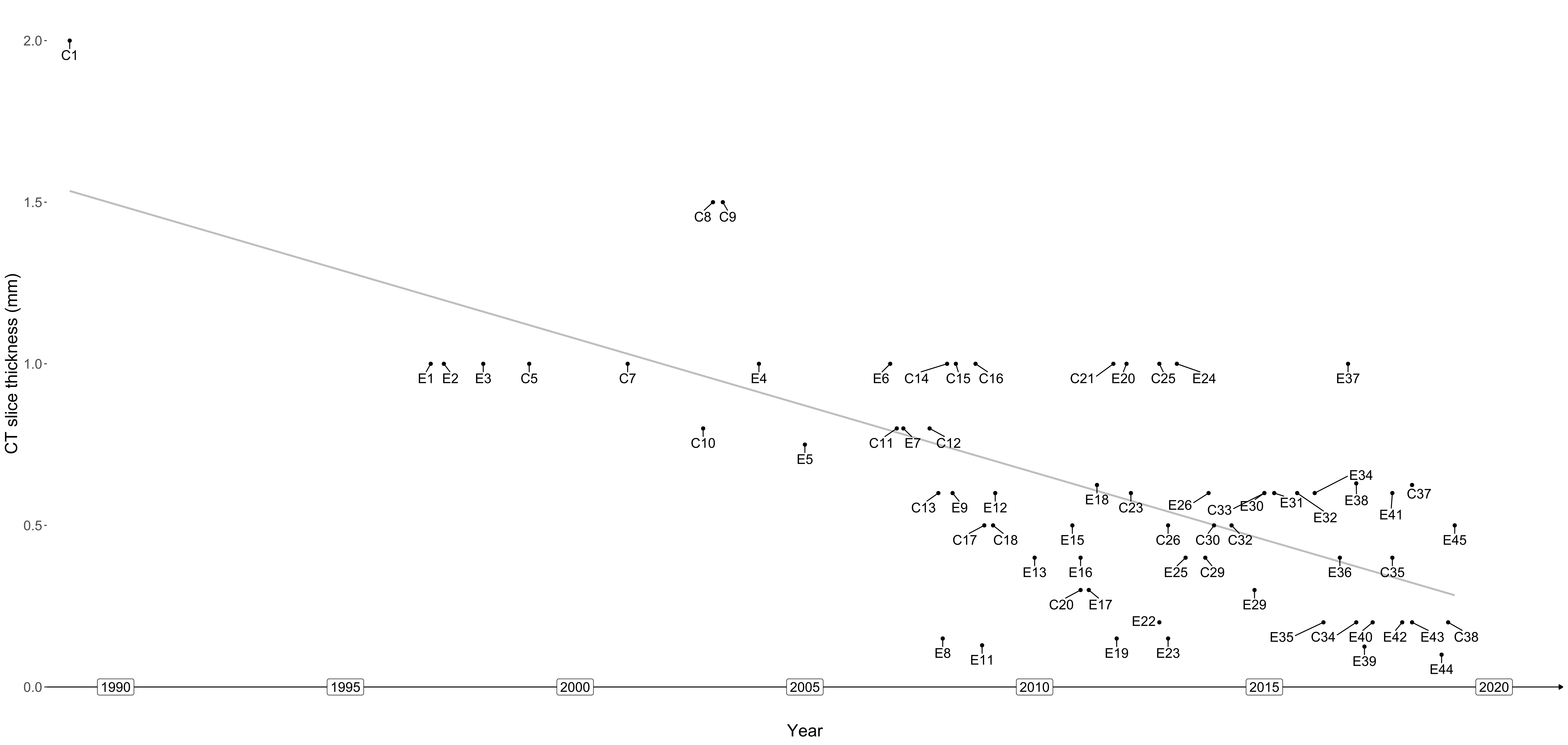
CT slice thickness (mm)
2.0
1.5
1.0
0.5
0.0
1990
1995
2000
2005
2010
2015
2020
Year
C1
C8 C9
E1 E2 E3 C5 C7 E4 E6 C14 C15 C16 C21 E20 C25 E24 E37
C10 E5 C11 E7 C12
C13 E9 E12 E18 C23 E26 C33 E30 E31 E32 E34 E38 E41 C37
C17 C18 E15 C26 C30 C32 E45
E13 E16 E25 C29 E36 C35
C20 E17 E29
E22 E35 C34 E40 E42 E43 C38
E8 E11 E19 E23 E39 E44
