# Supplementary material for: Evolution and Stagnation of Image Guidance for Surgery in the Lateral Skull: A Systematic Review 1989–2020
Source: Front Surg. 2021 Jan 11;7:604362. doi: 10.3389/fsurg.2020.604362 (PMC7831154; doi:10.3389/fsurg.2020.604362)
Supplement: Supplementary file 3 [file Data_Sheet_3.DOCX]

(((Surgery, Computer-Assisted[MeSH Terms]) OR Neuronavigation[MeSH Terms]) OR Stereotaxic Techniques[MeSH Terms])

AND (Cadaver[MeSH Terms] OR Humans[MeSH Terms])

AND ((((((Cranial Fossa, Middle[MeSH Terms]) OR Cranial Fossa, Posterior[MeSH Terms]) OR Otolaryngology[MeSH Terms]) OR Temporal Bone[MeSH Terms]) OR Otologic Surgical Procedures[MeSH Terms]) OR lateral skull base[Title/Abstract])

NOT (((((((*endonas*[Title/Abstract]) OR *sinus*[Title/Abstract]) OR *nasal*[Title/Abstract]) OR *oral*[Title/Abstract]) OR *orbit*[Title/Abstract]) OR paranasal sinuses[MeSH Terms]) OR Radiotherapy[MeSH Terms])

NOT Review[Publication Type]
